# *ANGPTL8* protein-truncating variant associated with lower serum triglycerides and risk of coronary disease

**DOI:** 10.1371/journal.pgen.1009501

**Published:** 2021-04-28

**Authors:** Pyry Helkkula, Tuomo Kiiskinen, Aki S. Havulinna, Juha Karjalainen, Seppo Koskinen, Veikko Salomaa, Mark J. Daly, Aarno Palotie, Ida Surakka, Samuli Ripatti, FinnGen

**Affiliations:** 1 Institute for Molecular Medicine Finland (FIMM), HiLIFE, University of Helsinki, Helsinki, Finland; 2 Finnish Institute for Health and Welfare, Helsinki, Finland; 3 Analytic and Translational Genetics Unit, Massachusetts General Hospital and Harvard Medical School, Boston, Massachusetts, United States of America; 4 Broad Institute of the Massachusetts Institute of Technology and Harvard University, Cambridge, Massachusetts, United States of America; 5 Psychiatric & Neurodevelopmental Genetics Unit, Department of Psychiatry, Analytic and Translational Genetics Unit, Department of Medicine, and the Department of Neurology, Massachusetts General Hospital, Boston, Massachusetts, United States of America; 6 Department of Internal Medicine, University of Michigan, Ann Arbor, Michigan, United States of America; 7 Department of Public Health, University of Helsinki, Helsinki, Finland; University of Pennsylvania Perelman School of Medicine, UNITED STATES

## Abstract

Protein-truncating variants (PTVs) affecting dyslipidemia risk may point to therapeutic targets for cardiometabolic disease. Our objective was to identify PTVs that were associated with both lipid levels and the risk of coronary artery disease (CAD) or type 2 diabetes (T2D) and assess their possible associations with risks of other diseases. To achieve this aim, we leveraged the enrichment of PTVs in the Finnish population and tested the association of low-frequency PTVs in 1,209 genes with serum lipid levels in the Finrisk Study (n = 23,435). We then tested which of the lipid-associated PTVs were also associated with the risks of T2D or CAD, as well as 2,683 disease endpoints curated in the FinnGen Study (n = 218,792). Two PTVs were associated with both lipid levels and the risk of CAD or T2D: triglyceride-lowering variants in *ANGPTL8* (-24.0[-30.4 to -16.9] mg/dL per rs760351239-T allele, *P* = 3.4 × 10^−9^) and *ANGPTL4* (-14.4[-18.6 to -9.8] mg/dL per rs746226153-G allele, *P* = 4.3 × 10^−9^). The risk of T2D was lower in carriers of the *ANGPTL4* PTV (OR = 0.70[0.60–0.81], *P* = 2.2 × 10^−6^) than noncarriers. The odds of CAD were 47% lower in carriers of a PTV in *ANGPTL8* (OR = 0.53[0.37–0.76], *P* = 4.5 × 10^−4^) than noncarriers. Finally, the phenome-wide scan of the *ANGPTL8* PTV showed that the *ANGPTL8* PTV carriers were less likely to use statin therapy (68,782 cases, OR = 0.52[0.40–0.68], *P* = 1.7 × 10^−6^) compared to noncarriers. Our findings provide genetic evidence of potential long-term efficacy and safety of therapeutic targeting of dyslipidemias.

## Author summary

Studying the health impacts of protein-truncating variants (PTVs) enables detecting the health impact of drugs that inhibit these same genes. Our study aimed to expand our knowledge of genes associated with cardiometabolic disease, along with the side effects of these genes. To detect PTVs associated with cardiometabolic disease, we first performed a genome-wide scan of PTVs associated with serum lipid levels in Finns. We found PTVs in two genes highly enriched in Finns, which were associated with both serum lipid levels and a lower risk of type 2 diabetes or coronary artery disease: *ANGPTL4* and *ANGPTL8*. To evaluate the other health effects of these PTVs, we performed an association scan between the PTVs and 2,683 disease endpoints curated in the FinnGen Study (n = 218,792). We demonstrate that using human populations with PTV-enrichment, such as Finns, offers considerable boosts in statistical power to detect potential long-term efficacy and safety of pharmacologically targeting genes.

## Introduction

Dyslipidemia is a major risk factor for cardiovascular disease and is present in nearly half of type 2 diabetes patients [1]. For treating dyslipidemia, there are few alternatives to low-density lipoprotein (LDL) cholesterol-lowering therapy. Although common, this therapy often fails to treat the condition effectively, leaving patients with high risk of cardiovascular disease [2]. Therefore, a search for possible new drugs is necessary. Although genome-wide association studies have identified over 200 genetic loci that are related to circulating lipid levels [3–6] these variants are typically common (minor-allele frequency [MAF] greater than 5%) and are located in the noncoding part of the genome. This has made it hard to identify causal genes for blood lipid levels and thus cardiometabolic disease risk for most genetic regions. Recent surveys on the protein-coding variation in lipid-associated loci [4,5,7] have implicated likely causal genes via low-frequency coding variants. However, these types of studies have not been able to show which genes can be pharmacologically inhibited safely to reduce the risk of type 2 diabetes (T2D) or coronary artery disease (CAD).

There is a limited number of drugs for treating dyslipidemia currently on the market or in development. Drugs targeting triglycerides, lipoprotein(a) or high-density lipoprotein (HDL) cholesterol that are currently being developed have emerged from studies of PTVs [8–14], and it is uncertain whether they are safe enough to reach the market. Meanwhile, PCSK9 inhibitors and ezetimibe are the only common alternatives to statins for lowering LDL cholesterol levels. More options would be welcome given that statins have common side effects [15].

Besides the small number of alternatives, another issue is the long-term safety of drugs targeting dyslipidemia. Maximizing the long-term safety of these drugs is important because they are often used preventatively and for prolonged periods by wide sectors of the population. Given this use, the long-term, population-level side effects of these drugs are especially important to minimize. Such an understanding could be achieved by investigating health impacts of genetic proxies for protein deficiencies that are associated with lipid levels. A study assessing this question exists for the chief lipoprotein(a)-modulating gene *LPA* [16]. However, it is less well known what are the health effects of the other major lipid-modifying genes targeted by drugs currently under development: *ANGPTL3*, *ANGPTL4*, *APOC3* and *CETP* [8–13]. Although some of these drug targets have undergone clinical trials, they do not consider long-term health impacts and suffer from confounding factors, such as compound-specific off-target effects.

Considering these clinical needs, the present study aimed to further the treatment of dyslipidemia in two ways. The primary goal was to identify PTVs associated with serum lipid levels and the risk of T2D or CAD, as well as assess their associations with other disease risks. Because of the clear deleterious effect of PTVs on the protein levels, studying them makes it possible to determine the effect of individual genes on disease risks and phenotypes over long time periods. Ultimately, such results provide an opportunity to develop safe drug targets for treating dyslipidemia and cardiometabolic diseases. The secondary aim of this study was to evaluate the long-term health consequences of existing dyslipidemia drug targets with the help of PTVs. Studying Finns provides a promising avenue for reaching both of these goals. The Finnish population isolate shows an enrichment of protein-truncating variants (PTVs)[17,18] (stop-gained, frame-shift and essential splice-site mutations), thus enabling the detection of both new and previously-known therapeutic effects through a smaller sample size than in the non-Finnish European population. We restricted the study to low-frequency and rare PTVs (MAF between 0.1% and 5%). On the one hand, associations with common variants (MAF > 5%) would have already been detected by past large consortium efforts. On the other hand, for very rare variants (MAF < 0.1%), our study would have had both insufficient imputation accuracy and statistical power to detect significant associations. Thanks to national health records on Finns, it is also possible to screen for a wide range of long-term health impacts associated with these PTVs. When combined, the data on PTVs and health records provide us with exactly the type of long-term, population-wide, on-target side effect data that is currently lacking, as discussed above.

## Results

**Fig 1:**
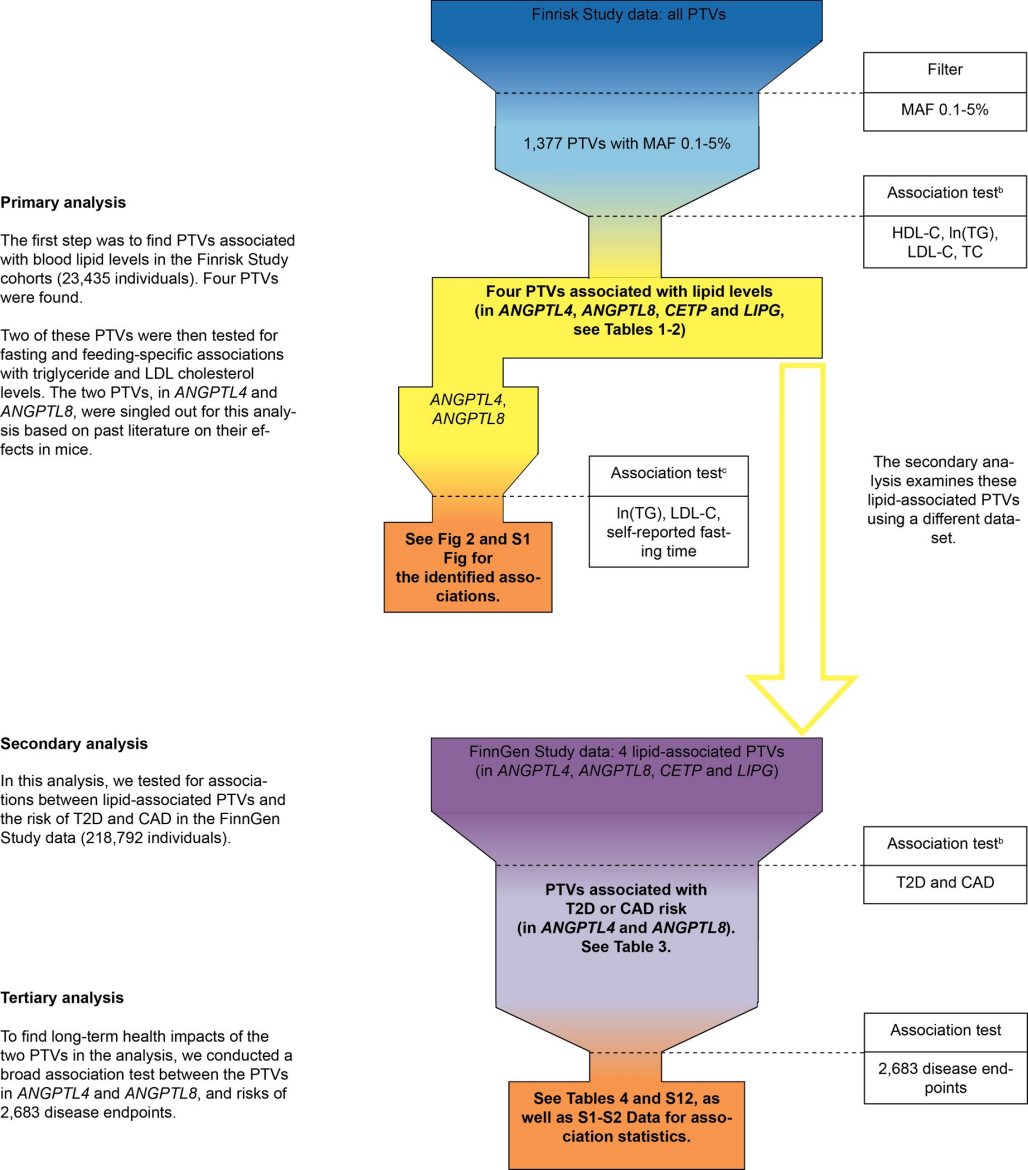
An overview of the present study. ^**a**^ Abbreviations: PTV, protein-truncating variant; MAF, minor-allele frequency; HDL-C, high-density lipoprotein cholesterol level; ln(TG), natural logarithm of triglyceride level; LDL-C, low-density lipoprotein cholesterol level; TC, total cholesterol level; T2D, type 2 diabetes; CAD, coronary artery disease. ^a^ Shown in the figure is an overview of the present study. The final results are shown in boxes with a bolded font. ^b^ Results for these association tests were also compared with other PTVs in UK Biobank. ^c^ These association tests were post-hoc in nature.

An overview of our study’s analyses and their results is presented in Fig 1. Our results are divided into three parts: the primary, secondary and tertiary analyses. Results of the primary analysis concern associations between 1,377 PTVs with a MAF of 0.1–5% and the serum lipid levels: triglycerides, LDL cholesterol, HDL cholesterol and total cholesterol. Results of the secondary analysis concern associations between lipid-associated PTVs and the risk of CAD and T2D. Finally, results of the tertiary analyses concern the possible side effects of PTVs associated with both lipid levels and the risk of T2D or CAD.

### Primary analyses

#### Protein-truncating variants and serum lipid levels

Through a genome-wide association scan between 1,377 PTVs and serum lipid levels we found four PTVs to be associated with serum lipid levels in 23,435 Finrisk Study individuals. PTVs in *CETP* (rs751916721-T), *LIPG* (rs200435657-A), and *ANGPTL8* (rs760351239-T) showed genome-wide significant associations with HDL cholesterol levels. PTVs in *ANGPTL4* (rs746226153-G) and *ANGPTL8* (rs760351239-T) had genome-wide significant associations with triglyceride levels (Tables 1 and 2). These observations are supported by previous studies. The splice acceptor variant rs200435657-A in *LIPG* and the stop-gained variant rs145464906-T in *ANGPTL8* have been associated genome-wide significantly with higher HDL cholesterol levels [4,19]. Nominal associations (*P* < 0.05) between lower triglyceride levels and protein-truncating variation in *ANGPTL8*[19] and *ANGPTL4*[9], as well as between *CETP* protein-truncating variation and higher HDL cholesterol levels [20], have also been reported. The four PTVs showed 27 to 210-fold enrichment in Finns compared to non-Finnish Europeans in the gnomAD database [21], version 2.1.1 (gnomad.broadinstitute.org) (Table 1).

**Table 1:** Associations between protein-truncating variants and serum lipid levels in the Finrisk Study.^a^

Locus and variant	Chromosome position^b^	Type of mutation	MAF in Finrisk Study *%*	MAF in NFE in gnomAD %	Enrichment^c^	Primary association	*P*	Conditional *P*^d^
*ANGPTL4 rs746226153-G*	19:8364556	Frameshift	0.48	1.9 × 10^−2^	27.2	Triglycerides	4.3 × 10^−9^	3.7 × 10^−8^
*ANGPTL8 rs760351239-T*	19:11240228	Stop-gained	0.15	2.1 × 10^−3^	85.0	Triglycerides	3.4 × 10^−9^	-
*CETP rs751916721-T*	16:56962097	Splice-site	0.12	3.1 × 10^−3^	46.3	HDL cholesterol	1.6 × 10^−21^	5.9 × 10^−19^
*LIPG rs200435657-A*	18:49565316	Splice-site	0.20	7.8 × 10^−4^	210.1	HDL cholesterol	5.0 × 10^−13^	3.6 × 10^−12^

Abbreviations: MAF, minor-allele frequency; NFE, non-Finnish Europeans.

^a^ Shown in the table are the four PTVs associated genome-wide significantly (two-sided *P* < 5 × 10^−8^) with at least one serum lipid level measure in the Finrisk Study cohorts. The tested serum lipid levels were LDL, HDL and total cholesterol, as well as natural logarithm transformed triglycerides.

^b^ Chromosome numbers and positions refer to genome build GRCh38.

^c^ The allele frequency enrichment in Finns with respect to non-Finnish Europeans according to the gnomAD database, version 2.1.1 (gnomad.broadinstitute.org).

^d^ The conditional P value is the largest P value from association tests conditioning on other previously reported genome-widely significant markers in the same gene. The conditional P value for rs760351239-T is not available because no genome-widely significant genetic variant in *ANGPTL8* has been reported before.

**Table 2:** Associations between protein-truncating variants and serum lipid levels in the Finrisk Study and UK Biobank.^a^

Locus and variant	Cohort	Triglycerides		LDL cholesterol		HDL cholesterol		Total cholesterol	
		Effect (95% CI)^b^ mg/dL	*P*	Effect (95% CI)^b^ mg/dL	*P*	Effect (95% CI)^b^ mg/dL	*P*	Effect (95% CI)^b^ mg/dL	*P*
*ANGPTL4 rs746226153-G*	Finrisk	-14.4 (-18.6 to -9.8)	4.3 × 10^−9^	2.4 (-2.1 to 7.0)	0.30	1.5 (-0.2 to 3.3)	0.09	-0.2 (-5.3 to 4.9)	0.94
*ANGPTL8 rs760351239-T*	Finrisk	-24.0 (-30.4 to -16.9)	3.4 × 10^−9^	-16.8 (-24.8 to -8.7)	4.5 × 10^−5^	9.1 (6.1 to 12.3)	4.6 × 10^−9^	-14.2 (-23.2 to -5.1)	2.2 × 10^−3^
*ANGPTL8 rs145464906-T*	UK Biobank	-18.9 (-21.2 to -15.1)	3.3 × 10^−25^	-4.5 (-7.6 to -1.4)	4.2 × 10^−3^	6.1 (4.8 to 7.4)	7.4 × 10^−20^	-1.0 (-5.2 to 3.1)	0.62
*CETP* *rs751916721-T*	Finrisk	5.1 (-5.2 to 16.8)	0.34	-7.0 (-16.1 to 2.1)	0.13	16.7 (13.2 to 20.1)	1.6 × 10^−21^	12.0 (1.9 to 22.2)	0.02
*LIPG* *rs200435657-A*	Finrisk	3.7 (-4.6 to 12.8)	0.40	4.1 (-3.4 to 11.7)	0.28	10.2 (7.5 to 13.0)	5.0 × 10^−13^	17.3 (9.0 to 25.5)	4.2 × 10^−5^

Abbreviations: CI, confidence interval.

^a^ All association tests in were adjusted for age, age squared, sex. In the Finrisk Study and UK Biobank association tests were additionally adjusted for the study cohort and ten principal components, and assessment center and 40 principal components respectively. In analyses of serum triglycerides, the triglyceride levels were natural logarithm transformed and waist-to-hip ratio was included as an additional covariate. Values for LDL and total cholesterol were adjusted for the use of lipid-lowering medication by dividing by 0.7 and 0.8 respectively.

^b^ The effect is the difference per each copy of the minor-allele in units in mg/dL of triglycerides, LDL, HDL and total cholesterol. To convert the values for cholesterol to mmol/L, multiply by 0.0259. To convert the values for triglycerides to mmol/L, multiply by 0.0113.

To evaluate whether the associations between the serum lipid levels and the four PTVs were independent, we conducted conditional tests of independence and determined which credible sets the PTVs belonged to. In our conditional analysis, the associations between lipid levels and the PTVs in the Finrisk Study [22] data were not explained by previously-reported genome-wide significant variants [3–6] (Tables 1 and S3–S6). The *LIPG* and *ANGPTL8* PTVs alone formed credible sets with posterior probabilities above 99.9%. The *CETP* and *ANGPTL4* PTVs were in high linkage disequilibrium with the noncoding variants rs566571297-T (*r*^2^ = 0.99) and rs919624228-G (*r*^2^ = 0.97) respectively. With their correlated variant pair, these two PTVs formed credible sets with posterior probabilities higher than 99%. Hence, all the four PTVs were independently associated with changes in lipid levels with a very high probability (S7–S10 Tables).

To inspect if the lipid level associations with PTVs in Finns were concordant with other PTVs in the same genes, we surveyed the UK Biobank data. Using another rare non-Finnish European-enriched *ANGPTL8* PTV (rs145464906-T) in Britons, we observed comparable and genome-wide significant HDL cholesterol and triglyceride level associations as with the PTV found in Finns (Table 2). PTVs in *ANGPTL4*, *CETP* or *LIPG* were not present in the UK Biobank data and thus we were not able to investigate the lipid level associations of other protein-truncating variation in these genes.

#### Protein-truncating variants and serum lipid levels as a function of fasting time

Angptl4 and Angptl8 have previously been linked to specific circulating lipid profiles in response to fasting and feeding respectively [23–25]. Therefore, we tested two hypotheses relating to the association of *ANGPTL4* and *ANGPTL8* with lipid levels as a function of fasting time. The first hypothesis was that *ANGPTL4* and *ANGPTL8* are associated with lower fasting and postprandial triglyceride levels respectively. We found that *ANGPTL4* PTV heterozygotes had 16.6% (20.2 mg/dL, *P* = 8.7 × 10^−7^) lower triglyceride levels than non-carriers when the time since the last meal was from 4 to 8 hours. On the other hand, the heterozygotes’ triglyceride levels were not significantly lower than non-carriers’ when the fasting time was 3 hours or less (14.8% higher [20.6 mg/dL], *P* = 0.09). The *ANGPTL8* PTV heterozygotes in turn had 34.3% (47.8 mg/dL, *P* = 0.01) lower triglyceride levels than non-carriers when the fasting time was up to 3 hours. From 4 to 8 hours after the last meal, the carriers had only 22.9% (27.9 mg/dL, *P* = 3.2 × 10^−4^) lower triglyceride levels than noncarriers.

**Fig 2:**
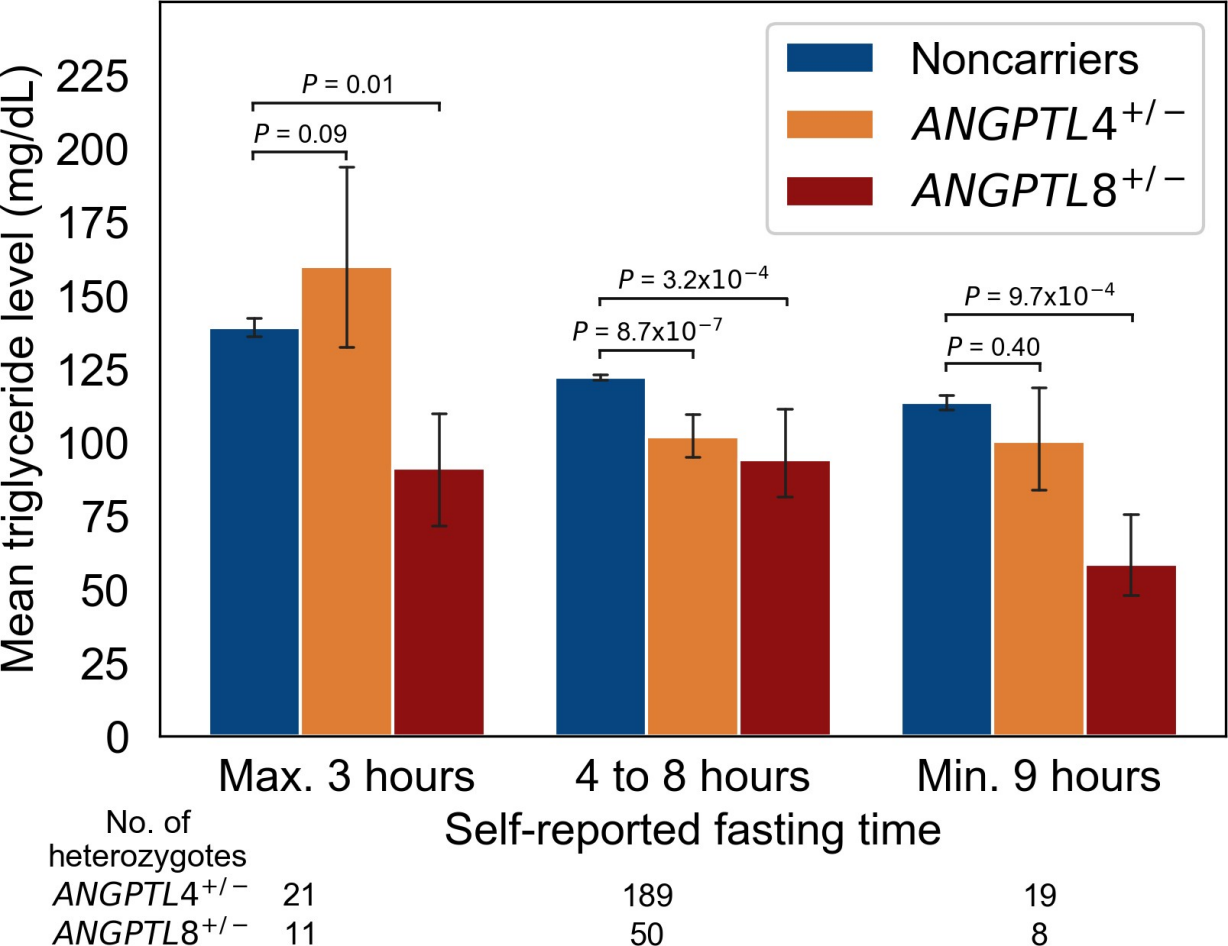
Mean serum triglyceride levels of *ANGPTL4* and *ANGPTL8* PTV heterozygotes at their self-reported fasting time in the Finrisk Study. ^**a** a^ The figure shows the mean serum triglyceride level by *ANGPTL4* and *ANGPTL8* PTV carrier status with respect to self-reported fasting time. The number of *ANGPTL4* and *ANGPTL8* heterozygotes for each fasting time interval are reported below the fasting time legend. The height of the bars indicate means and the error bars 95% confidence intervals. The P values are for the two-sided Welch’s t-test of the natural logarithm of triglyceride levels between noncarriers and heterozygotes.

The second hypothesis was that the *ANGPTL8* PTV is associated with lower fasting LDL cholesterol and triglyceride levels. We observed that *ANGPTL8* PTV heterozygotes had 13.4% (18.0 mg/dL, *P* = 4.5 × 10^−5^) lower LDL cholesterol levels than noncarriers with a fasting time between 4 and 8 hours. Moreover, the heterozygotes had 48.6% (55.1 mg/dL, *P* = 9.7 × 10^−4^) and 26.2% (36.9 mg/dL, *P* = 0.007) lower triglyceride and LDL cholesterol levels respectively than noncarriers when fasting for 9 hours or longer. The triglyceride and LDL cholesterol levels as a function of *ANGPTL4* and *ANGPTL8* PTV genotype and fasting time are shown in Figs 2 and S1 respectively.

### Secondary analyses

#### Lipid-associated protein-truncating variants and the risk of T2D and CAD

Given that the primary analysis suggested that *ANGPTL4* and *ANGPTL8* PTVs may reduce triglyceride levels, we tested these PTVs’ impact on type 2 diabetes (T2D), coronary artery disease (CAD) risk in 218,792 individuals from the FinnGen Study. The *ANGPTL8* PTV was associated (two-tailed *P* < 4.1 × 10^−3^) with lower odds of CAD (OR = 0.53[0.37–0.76], *P* = 4.5 × 10^−4^). The *ANGPTL4* PTV heterozygotes had 30% lower odds of T2D (OR = 0.70[0.60–0.81], *P* = 2.2 × 10^−6^). Finally, we tested if the four PTVs identified in our primary analysis were associated with available traditional non-lipid risk factors for CAD in the FinnGen Study (S11 Table). Briefly, *ANGPTL4*, *ANGPTL8* and *CETP* PTVs were associated with lower risks of using statin medication. *ANGPTL4* and *LIPG* PTV carriers in turn had lower risks of hypertension compared to noncarriers.

Finally, as in the primary analysis, we checked if our findings for the Finnish-enriched *ANGPTL8* PTV (rs760351239-T) were congruent with the other *ANGPTL8* PTV enriched in UK Biobank. In specific, we tested if the non-Finnish European-enriched *ANGPTL8* PTV (rs145464906-T) was associated with T2D and CAD risk. Although rs145464906-T in the UK Biobank data alone was not significantly associated with the risk of T2D or CAD, a meta-analysis of the rs760351239-T and rs145464906-T PTVs strengthened both of these associations (Table 3).

**Table 3:** Association between protein-truncating variants and T2D and CAD risk in the FinnGen Study and UK Biobank.^a^

Locus and variant	Cohort	Type 2 diabetes; 29,166 cases in FinnGen and 18,945 cases in UK Biobank				Coronary artery disease; 23,363 cases in FinnGen and 20,023 cases in UK Biobank			
		Allele frequency^b^		OR^c^ (95% CI)	*P*	Allele frequency^b^		OR^c^ (95% CI)	*P*
		Cases %	Controls %			Cases %	Controls %		
*ANGPTL4* *rs746226153-G*	FinnGen	0.41	0.53	0.70 (0.60–0.81)	2.2 × 10^−6^	0.51	0.51	0.94 (0.80–1.12)	0.50
*ANGPTL8* *rs760351239-T*	FinnGen	0.09	0.12	0.67 (0.49–0.93)	0.02	0.09	0.12	0.53 (0.37–0.76)	4.5 × 10^−4^
*ANGPTL8* *rs145464906-T*	UK Biobank	0.04	0.05	0.79 (0.48–1.30)	0.35	0.03	0.05	0.73 (0.44–1.19)	0.21
*ANGPTL8* PTV meta-analysis^d^	FinnGen and UK Biobank	0.06	0.07	0.70 (0.54–0.92)	0.01	0.05	0.07	0.59 (0.44–0.79)	3.3 × 10^−4^
*CETP* *rs751916721-T*	FinnGen	0.08	0.11	0.66 (0.48–0.92)	0.01	0.11	0.10	1.06 (0.73–1.53)	0.76
*LIPG* *rs200435657-A*	FinnGen	0.13	0.13	0.87 (0.65–1.15)	0.33	0.14	0.13	0.79 (0.58–1.09)	0.15

Abbreviations: T2D, type 2 diabetes; CAD, coronary artery disease; OR, odds ratio; CI, confidence interval.

^a^ Shown in the table are the association statistics between the serum lipid-associated PTVs and type 2 diabetes and coronary artery disease risk.

^b^ The allele frequencies are reported in percent. The rs145464906-T allele frequencies in cases and controls were calculated in 343,687 unrelated individuals in UK Biobank and are based on PheCodes [38] 250.2 and 411.4 for type 2 diabetes and coronary artery disease respectively.

^c^ In the FinnGen data the odds ratios were calculated using SAIGE [31] saddle-point approximation-based score test after adjustment for age, sex, genotyping batch and ten principal components of ancestry. The odds ratios for the associations between the disease risks and PTVs in UK Biobank were obtained from Zhou *et al*. [31]

^d^ The rs760351239-T and rs145464906-T PTVs present in FinnGen and UK Biobank genotype data respectively have been meta-analyzed

### Tertiary analyses

#### Phenome-wide associations of protein-truncating variants in *ANGPTL4* and *ANGPTL8*

To evaluate a wide range of possible long-term health effects of the PTVs associated with both lipid levels and the risk of T2D or CAD, we tested the association between these PTVs and 2,683 curated disease endpoints. In addition to T2D risk, the *ANGPTL4* PTV was phenome-wide significantly associated (two-tailed *P* < 1.8 × 10^−5^) with multiple T2D-related disease endpoints and comorbidities (S12 Table). The *ANGPTL8* PTV was phenome-wide significantly associated with 48% lower odds of statin therapy (OR = 0.52[0.40–0.68], *P* = 1.7 × 10^−6^) (Table 4). See Online Tables 1 and 2 for the complete phenome-wide association statistics of the PTVs.

**Table 4:** Phenome-wide associations with the *ANGPTL8* protein-truncating variant in the FinnGen Study.^a^

	*ANGPTL8* PTV rs760351239-T				
Phenotype	No. of cases	Allele frequency^b^		OR^c^ (95% CI)	*P*
		Cases %	Controls %		
Statin medication	68,782	0.09	0.12	0.52 (0.40–0.68)	1.7 × 10^−6^

Abbreviations: PTV, protein-truncating variant; OR, odds ratio; CI, confidence interval.

^a^ Shown in the table are the phenome-wide significant associations (*P* < 1.8 × 10^−5^) for the rs760351239-T allele in the FinnGen Study.

^b^ The allele frequencies are reported in percent.

^c^ Odds ratios were calculated using SAIGE [31] saddle-point approximation-based score test after adjustment for age, sex, genotyping batch and ten principal components of ancestry.

## Discussion

In this study, we first presented associations between serum lipid levels and PTVs in four genes; *ANGPTL4*, *ANGPTL8*, *CETP* and *LIPG*. Especially the *ANGPTL8* PTV carriers showed an overall improved serum lipid level profile compared to noncarriers: significantly lower triglyceride and higher HDL cholesterol levels but also suggestively lower LDL and total cholesterol levels. We then showed that carriers of PTVs in *ANGPTL4* and *ANGPTL8* had lower risk of T2D and CAD, respectively, than noncarriers. Finally, we showed that the PTVs in *ANGPTL4* and *ANGPTL8* were associated with lower risks of diabetes-related endpoints and statin therapy respectively.

Firstly, our study points to *ANGPTL8* as a potential new therapeutic target for lowering triglyceride levels. This is interesting because while *CETP*, *LIPG* and *ANGPTL4* are well-known lipid genes, a genome-wide significant association between a PTV in *ANGPTL8* and triglyceride levels has not previously been reported in humans. ANGPTL8 is known to inhibit triglyceride-hydrolyzing lipoprotein lipase [26,27]. In our data, the association between *ANGPTL8* PTV and lower triglyceride levels had the strongest effects soon after a meal in a post-prandial state, and after prolonged fasting. These findings are in line with animal studies which show that Angptl8 is secreted after feeding and has a very short half-life [23,24], and that hepatic very low-density lipoprotein (VLDL) secretion is decreased in *Angptl8* knockout mice [28].

Our results also show that carriers of a Finnish-enriched *ANGPTL8* PTV had 47% lower odds of CAD than noncarriers. This is in line with genetic evidence of triglycerides being a causal factor for CAD risk [29]. Examining another non-Finnish European-enriched *ANGPTL8* PTV (rs145464906-T) in the UK Biobank data, we observed that also this PTV was genome-wide significantly associated with lower triglyceride levels. In addition, a meta-analysis of the *ANGPTL8* PTVs in FinnGen and UK Biobank data increased the statistical significance of T2D and CAD risk associations observed in FinnGen data alone. On the whole, an improved lipid profile and lower risk of CAD in *ANGPTL8* PTV carriers compared to noncarriers lends support to the efficacy of an antibody-based inhibition of ANGPTL8 in treating dyslipidemia and CAD.

Our results concerning the link between *ANGPTL8* and T2D risk are less certain, but still contribute to the existing literature. A previous study by Clapham *et al*. tested for the association between the rs145464906-T variant and T2D risk but found no significant association [30]. In UK Biobank data, Zhou *et al*. reported an odds ratio of 0.79[31] which marks a non-zero effect, but a wide confidence interval (0.48–1.30), indicating insufficient statistical power. Lack of statistical power was also a challenge in our study: in the FinnGen data, the association between the Finnish-enriched *ANGPTL8* PTV was nominally significant (*P* < 0.05) but not after multiple-testing correction. However, the statistical power to detect an association between T2D risk and *ANGPTL8* protein-truncating variation was higher in the FinnGen data due to a considerably higher number of T2D cases (29,166) compared to both the previous study by Clapham *et al*. [30] (14,824 cases and 80,734 controls) and Zhou *et al*. [31] (18,945 cases 388,756 controls). To conclusively confirm the association between *ANGPTL8* protein-truncating variation and T2D risk, a dataset with an even higher statistical power is needed.

Lastly, in addition to this main contribution on the potential benefits of the *ANGPTL8* PTV, our study also found associations between the *ANGPTL4* PTV and a lower risk of T2D, as well as multiple diabetes-related disease endpoints. An earlier report also found that *ANGPTL4* PTV carriers had a lower risk of T2D than noncarriers [32]. Also, a low-frequency missense variant in *ANGPTL4* has been associated with T2D and CAD risk [3,4].

Our study also has some limitations. Firstly, our study was observational in nature and consequently unable to directly reveal causal effects between genes and outcomes. The results of the tests for conditional variant association and credible sets do however increase the probability that the shortlisted PTVs indeed are causal variants underlying the observed association due to their statistical independence. The *ANGPTL8* and *LIPG* PTVs formed their respective 95% credible sets alone and the *ANGPTL4* and *CETP* in turn with only one other non-coding variant in high linkage disequilibrium with the respective PTV. Additional support for the robust lipid-level associations observed in this study comes from previous publications or replications using other high-confidence PTVs. Associations between lipid levels and the *LIPG*, *CETP* and *ANGPTL4* PTVs using the same [4] or other high-confidence PTVs in the same gene [20,33] have been reported before. In the case of *ANGPTL8*, we replicated the associations between HDL cholesterol and triglyceride levels using the high-confidence PTV rs145464906-T present in the UK Biobank data. Despite a replication using the rs145464906-T, a formal replication of the Finnish-enriched rs760351239-T using the same variant is warranted. A formal replication using exactly the same variant was unfeasible due to a combination of rarity, regionality and Finnish population specificity of rs760351239-T. Nevertheless, to confirm the causality between the associated disease risks and the Finnish-enriched PTVs, our findings need to be studied using human cell lines. Secondly, while utilizing samples from the bottlenecked Finnish population offered us considerable boosts in statistical power to test Finnish-enriched variants compared to samples from non-bottlenecked populations, variants at lower frequencies in Finns than other populations lack this particular boost.

In summary, we identified PTVs in *ANGPTL4* and *ANGPTL8* that were associated with lower triglyceride levels and PTVs in *CETP* and *LIPG* that were associated with higher HDL cholesterol levels. The carriers of PTVs in *ANGPTL4* and *ANGPTL8* had lower risks of T2D and CAD respectively. These findings point to potential target genes for development of novel preventive medication against T2D and CAD and highlight the utility of bottleneck populations in search of associations between protein-truncating variation and biomarkers.

## Methods

### Ethics statement

All participants gave written informed study-specific consent. Patients and control subjects in the FinnGen Study provided informed consent for biobank research, based on the Finnish Biobank Act. Alternatively, older Finnish research cohorts, collected prior the start of FinnGen Study (August 2017), were collected based on study-specific consents and later transferred to the Finnish biobanks after approval by Fimea, the National Supervisory Authority for Welfare and Health. Recruitment protocols followed the biobank protocols approved by Fimea. The Coordinating Ethics Committee of the Hospital District of Helsinki and Uusimaa (HUS) approved the FinnGen Study protocol No. HUS/990/2017.

The FinnGen project is approved by Finnish Institute for Health and Welfare (THL), approval number THL/2031/6.02.00/2017, amendments THL/1101/5.05.00/2017, THL/341/6.02.00/2018, THL/2222/6.02.00/2018, THL/283/6.02.00/2019, THL/1721/5.05.00/2019), Digital and population data service agency VRK43431/2017-3, VRK/6909/2018-3, VRK/4415/2019-3, the Social Insurance Institution (KELA) KELA 58/522/2017, KELA 131/522/2018, KELA 70/522/2019, KELA 98/522/2019, and Statistics Finland TK-53-1041-17.

The Biobank Access Decisions for FinnGen Study samples and data utilized in FinnGen Data Freeze 5 include: THL Biobank BB2017_55, BB2017_111, BB2018_19, BB_2018_34, BB_2018_67, BB2018_71, BB2019_7, BB2019_8, BB2019_26, Finnish Red Cross Blood Service Biobank 7.12.2017, Helsinki Biobank HUS/359/2017, Auria Biobank AB17-5154, Biobank Borealis of Northern Finland_2017_1013, Biobank of Eastern Finland 1186/2018, Finnish Clinical Biobank Tampere MH0004, Central Finland Biobank 1–2017, and Terveystalo Biobank STB 2018001.

### Overview of the study

We identified PTVs associated with both lipid levels and the risk of T2D or CAD, and then examined their associations with other disease endpoints. In our primary analysis, we studied 23,435 Finns to find PTVs associated with serum lipid levels. Using data from the FinnGen Study, which is based on 218,792 individuals, we then studied the association between these mutations and the risk of T2D and CAD. We refer to this as our secondary analysis. In our tertiary and final analysis, using FinnGen data, we assessed the long-term health impacts of the PTVs’ associations with T2D and CAD risk by screening them for modified risk of 2,683 diseases. An overview of our study is depicted in Fig 1.

### Study populations

We used three different data sets for our study: the Finrisk Study cohorts, the FinnGen Study and UK Biobank. In total, the Finrisk Study dataset contained 23,435 chip-genotyped and imputed samples, selected randomly from the Finnish population in 1992, 1997, 2002, 2007 and 2012[22]. The first and second-degree relatives were limited in the set by limiting the identity by descent to 25% (the —rel-cutoff option set to 0.25 in PLINK). The baseline characteristics of the Finrisk Study participants are shown in S1 Table.

The FinnGen Study contains biobank data and national health registry data for 218,792 individuals. The health registry information of participants from the Finrisk and FinnGen Study was followed up until 31.12.2018. The details of the individual FinnGen cohorts are shown in S2 Table. All Finrisk and FinnGen Study participants were of Finnish descent. The genotyping and imputation data release of UK Biobank data was from 5th March 2018 and included 343,687 unrelated white British individuals.

### Genotyping and quality control

The Finrisk Study samples were genotyped using the HumanCoreExome BeadChip, Human610-Quad BeadChip, Affymetrix6.0 and Infinium HumanOmniExpress (Illumina Inc., San Diego, CA, USA) chips and a Finnish-ancestry-specific imputation panel consisting of 2,690 deep-coverage (25-30x) whole-genome and 5,092 whole-exome sequences. In the primary analysis, the 1,377 PTVs (stop-gained, frameshift and essential splice-site mutations) were located in 1,209 genes and had a MAF between 0.1 and 5%. These PTVs in the Finrisk Study cohorts were imputed and had a IMPUTE2[34] genotype information score with a mean of 0.95 (standard deviation of 0.05) and a minimum of 0.75. The FinnGen Study samples were genotyped with various Illumina and a custom AxiomGT1 Affymetrix array (www.finngen.fi/en/researchers/genotyping). All the lipid-associated PTVs were directly genotyped in at least 70.0% of the FinnGen Study individuals with the AxiomGT1 Affymetrix array. The genotypes of the PTVs in our secondary and tertiary analyses that were not genotyped on chip were imputed using a genotype panel that consisted of 3,775 deep-coverage (25-30x) whole-genome sequenced individuals of Finnish ancestry. The PTVs in our secondary and tertiary analyses had IMPUTE2[34] genotype information scores above 0.93 in the FinnGen data. Detailed description of the genotyping methods, genotype imputation and quality-control procedures are described in S2 Text.

For the association analyses between lipid levels and the rs145464906-T *ANGPTL8* PTV we included only white British individuals from UK Biobank and removed samples with excess heterozygosity or genotype missingness, sex chromosome aneuploidies and a mismatch between inferred and reported sex. Finally, related individuals were removed by limiting KING’s (http://people.virginia.edu/~wc9c/KING/) [35] kinship value to 0.0442.

### Study outcomes

A blood sample and the self-reported fasting time since the previous meal at the time of blood sample collection of each Finrisk Study participant were collected during a clinical visit. The total cholesterol, HDL cholesterol and triglyceride levels were measured directly from serum or plasma and LDL cholesterol was either directly measured or estimated using the Friedewald formula [36]. In UK Biobank the blood lipid levels were measured from serum directly. LDL and total cholesterol levels of individuals with lipid-lowering therapy were divided by 0.7 and 0.8 [37] respectively.

Information on diagnoses in the FinnGen data were collected and confirmed by examining national healthcare registries and recorded using the *International Classification of Diseases* [ICD] revisions 8–10. Purchase information on prescription drugs since 1995 were obtained from the Finnish social insurance institution (KELA) reimbursement records and coded using the *Anatomical Therapeutic Chemical* [ATC] classification). All FinnGen Study participants’ healthcare registry information were followed up until 31.12 2017. Cancer diagnoses and causes of death were obtained from their respective national registries. The clinical expert groups of the FinnGen Study have defined disease events using ICD and ATC codes. For a complete list of the considered clinical endpoints and corresponding ICD and ATC codes, see the link: www.finngen.fi/en/researchers/clinical-endpoints. For the T2D and CAD statuses in UK Biobank participants we used PheCodes [38] 250.2 and 411.4 respectively.

### Study design and statistical analyses

#### Primary analysis

In this analysis, we sought to identify associations between lipid levels and PTVs in the Finrisk Study. The lipid levels tested were plasma or serum levels of HDL cholesterol, LDL cholesterol, total cholesterol and logarithmically transformed triglycerides on a natural log scale. Our model was additive, and included age, age squared, sex, collection year, and ten principal components of ancestry as fixed-effects covariates. To correct for the effect of adiposity on triglyceride levels, we adjusted the triglyceride association tests for waist-to-hip ratio as well. We considered the genome-wide association significance threshold of a two-sided P value of less than 5.0 × 10^−8^ to be significant. Genetic association analyses were carried out using the PLINK [39], version v1.90b3.45 (www.cog-genomics.org/plink/1.9/), file format, Python, version 3.6 (www.python.org) and the statsmodels Python package, version 0.8.0 (www.statsmodels.org). In our scan, we only considered variants with a MAF 0.1–5% to account for adequate statistical power and the expected low frequency of high-impact alleles. To assess the statistical independence of the associations, we performed conditional analyses with previously associated variants [3–6] (Table 1 and S3–S6 Tables) and determined the 95% credible sets of variants in each gene locus with a 1 Mb window (S7–S10 Tables). The credible sets were determined using FINEMAP [40], version 1.4.3 (www.finemap.me).

Next, we carried out a post-hoc analysis to test the hypotheses that *ANGPTL4* and *ANGPTL8* PTVs are associated with triglycerides and LDL cholesterol levels as a function of fasting time. These hypotheses were based on animal studies that we reviewed after finding four PTVs associated with lipids in our genome-wide analysis. Firstly, we found studies that showed that in mice, Angptl4 and Angptl8 inhibit lipoprotein lipase (LPL) as a function of fasting time [23–25]. LPL inhibition is important because it is the mechanism by which several triglyceride-lowering drugs currently under development work [8,9,12]. If the association between triglyceride levels and *ANGPTL8* and *ANGPTL4* PTVs depends on fasting time, then fasting time dependent effects on triglyceride levels of these PTVs are very relevant for our study, which aims to assess the effect of these PTVs on hypertriglyceridemia risk.

Literature similarly suggested that, to determine whether *ANGPTL8* is a viable drug target, it might also be important to test its association with LDL cholesterol levels as a function of fasting time. A mouse study found that *ANGPTL8* modulates VLDL secretion [28]. Given our goal of testing the viability of *ANGPTL8* as a drug target, the effect of *ANGPTL8* on VLDL levels would be important to assess in humans because high VLDL levels are associated with a higher risk of CAD [41]. We could not directly test whether human *ANGPTL8* PTV carriers had lower VLDL levels than noncarriers, so we instead tested the association between the *ANGPTL8* PTV and levels of LDL cholesterol and triglycerides after fasting. Our rationale for this test was that *fasting* triglyceride and LDL cholesterol levels in particular can be considered a proxy for VLDL. This is due to the deficiency of chylomicron and chylomicron remnant particles in the bloodstream in a fasted state [42].

We examined the serum lipid level associations of other PTVs in the same genes as in our findings, using UK Biobank data. For this we used another rare non-Finnish European-enriched *ANGPTL8* PTV (rs145464906-T). PTVs in *ANGPTL4*, *CETP* or *LIPG* were not present in the UK Biobank data and thus we were not able to analyze the lipid level associations of protein-truncating variation in these genes.

#### Secondary and tertiary analyses

In the secondary analyses, we examined associations between the risk of T2D and CAD, and the lipid-associated PTVs identified in the primary analysis. The associations were tested on data from the FinnGen Study and we regarded a two-sided P value below 4.1 × 10^−3^ (Bonferroni-corrected threshold for 12 tests) to be statistically significant.

As in the primary analysis, we checked if our findings for the Finnish-enriched *ANGPTL8* PTV (rs760351239-T) were consistent with another rare *ANGPTL8* PTV in UK Biobank. We tested if the non-Finnish European-enriched *ANGPTL8* PTV (rs145464906-T) was associated with T2D and CAD risk. In addition, we conducted an inverse-variance-weighted meta-analysis of the rs760351239-T and rs145464906-T PTVs in the FinnGen and UK Biobank data.

In the tertiary analyses we examined the other health impacts of the PTVs associated with both serum lipid levels and the risk of T2D or CAD. We screened these PTVs broadly for modified risk of 2,683 diseases in the FinnGen data. We regarded a two-sided P value below 1.8 × 10^−5^ (Bonferroni-corrected threshold for 2,683 traits) to be statistically significant.

In both the secondary and tertiary analyses in the FinnGen data, the odds ratios for disease outcomes were estimated using SAIGE [31], version 0.35.8.8 (www.github.com/weizhouUMICH/SAIGE/releases/tag/0.35.8.8). Age, sex, genotyping batch and ten principal components of ancestry and the kinship matrix were included as fixed-effects covariates. See S1–S2 Data for the association statistics between disease risks and the *ANGPTL4* and *ANGPTL8* PTVs and the link: https://www.finngen.fi/en/researchers/clinical-endpoints for disease endpoint definitions in FinnGen. The details of computing the associations in the FinnGen data are described in the Supporting information. The T2D and CAD risk associations were also computed using SAIGE and were obtained from Zhou *et al*. [31].

## Supporting information

S1 Text List of FinnGen Study members. People who contributed to the FinnGen Study are listed in the supplementary text.
(PDF)

S2 Text Supplementary methods. A detailed description of the measures used in genotyping, quality control and statistical analyses of the Finrisk and FinnGen Study samples is included in this document.
(DOCX)

S1 Table Baseline characteristics of the 23,435 Finrisk Study participants.^**a**^ ^a^ Shown in the table are the baseline characteristics of the Finrisk Study participants used for finding the PTVs associated with blood lipid levels.
(XLSX)

S2 Table The FinnGen Study samples data release 5, version 1.0.^**a**^ ^a^ AxiomGT1 batches 9 and 11 had possibly contaminated samples identified by excessive relatedness (pihat linkage cutoff ≥ 0.1 for more than 30 samples). Therefore, in the QC step before imputation, an additional 83 and 50 samples were removed from batches 9 and 11 respectively.
(XLSX)

S3 Table Joint testing of previously associated variants in the *CETP* locus, rs751916721-T and high-density lipoprotein cholesterol levels.^**a**^ ^a^ This table shows the association statistics in Finrisk Study data of previously reported genome-wide associations in the *CETP* locus and the association statistics when the rs751916721-T variant is tested in a joint model with the earlier associations. Missing results are due to the rarity of a previously known variant in the Finrisk genotype data. ^b^ rs821840 is located in a low complexity region and was thus excluded from the Finrisk genotype data; hence the missing data.
(XLSX)

S4 Table Joint testing of previously associated variants in the *LIPG* locus, rs200435657-A and high-density lipoprotein cholesterol levels.^**a**^ ^a^ This table shows the association statistics in Finrisk Study data of previously reported genome-wide associations in the *LIPG* locus and the association statistics when the rs200435657-A variant is tested in a joint model with the earlier associations. Missing results are due to the rarity of a previously known variant in the Finrisk genotype data.
(XLSX)

S5 Table Joint testing of previously associated variants in the *ANGPTL4* locus, rs746226153-G and triglyceride levels.^**a**^ ^a^ This table shows the association statistics in Finrisk Study data of previously reported genome-wide associations in the *ANGPTL4* locus and the association statistics when the rs746226153-G variant is tested in a joint model with the earlier associations. Missing results are due to the rarity of a previously known variant in the Finrisk Study genotype data.
(XLSX)

S6 Table Joint testing of previously associated variants in the *ANGPTL8* locus, rs760351239-T and triglyceride levels.^**a**^ ^a^ This table shows the association statistics in Finrisk Study data of previously reported genome-wide associations in the *ANGPTL8* locus and the association statistics when the rs760351239-T variant is tested in a joint model with the earlier associations. Missing results are due to the rarity of a previously known variant in the Finrisk Study genotype data.
(XLSX)

S7 Table Top configuration of the variants forming the 95% credible sets and their probabilities in the *CETP* locus.^**a**^ ^a^ Shown in the table are the variants with the highest posterior probability in each 95% credible set in the *CETP* locus. The columns cred1-cred5 and prob1-prob5 indicate these variants and their respective posterior probabilities in each credible set. ^b^ The noncoding rs566571297 and protein-truncating rs751916721 variants are in very high linkage disequilibrium (*r*^2^ = 0.99).
(XLSX)

S8 Table Top configuration of the variants forming credible sets in the *LIPG* locus.^**a**^ ^a^ Shown in the table are the variants with the highest posterior probability in each 95% credible set in the *LIPG* locus. The columns cred1-cred4 and prob1-prob4 indicate these variants and their respective posterior probabilities in each credible set.
(XLSX)

S9 Table Top configuration of the variants forming credible sets in the *ANGPTL4* locus.^**a**^ ^a^ Shown in the table are the variants with the highest posterior probability in each 95% credible set in the *ANGPTL4* locus. The columns cred1-cred2 and prob1-prob2 indicate these variants and their respective posterior probabilities in each credible set. ^b^ The noncoding rs919624228 and protein-truncating rs746226153 variants are in very high linkage disequilibrium (*r*^2^ = 0.97).
(XLSX)

S10 Table Top configuration of the variants forming credible sets in the *ANGPTL8* locus.^**a**^ ^a^ Shown in the table are the variants with the highest posterior probability in each 95% credible set in the *ANGPTL8* locus. The columns cred1 and prob1 indicate the variant and its posterior probability in each credible set.
(XLSX)

S11 Table Associations between the lipid-associated protein-truncating variants, and hypertension and statin medication in the FinnGen Study.^**a**^ ^a^ Shown in the table are the associations between the lipid-associated PTVs, and hypertension and statin medication in the FinnGen Study. ^b^ The allele frequencies are reported in percent. ^c^ The ORs were calculated using SAIGE saddle-point approximation-based score test after adjustment for age, sex, genotyping batch and ten principal components of ancestry in the FinnGen Study.
(XLSX)

S12 Table Phenome-wide associations with the *ANGPTL4* protein-truncating variant in the FinnGen Study. ^**a**^ Abbreviations: PTV, protein-truncating variant; OR, odds ratio; CI, confidence interval. ^a^ Shown in the table are the phenome-wide significant associations (*P* < 1.8 × 10^−5^) for the rs746226153-G allele in the FinnGen Study. ^b^ The allele frequencies are reported in percent. ^c^ Odds ratios were calculated using SAIGE saddle-point approximation-based score test after adjustment for age, sex, genotyping batch and ten principal components of ancestry.
(XLSX)

S1 Data Association statistics between disease endpoints and the *ANGPTL4* PTV rs746226153-G in the FinnGen Study. For a complete list of the considered clinical endpoints and the corresponding ICD and ATC codes in the fifth data release of the FinnGen data see the link: www.finngen.fi/en/researchers/clinical-endpoints.
(XLSX)

S2 Data Association statistics between disease endpoints and the *ANGPTL8* PTV rs760351239-T in the FinnGen Study. For a complete list of the considered clinical endpoints and the corresponding ICD and ATC codes in the fifth data release of the FinnGen data see the link: www.finngen.fi/en/researchers/clinical-endpoints.
(XLSX)

S1 Fig Mean LDL cholesterol levels at the self-reported fasting time of *ANGPTL4* and *ANGPTL8* PTV heterozygotes, as well as noncarriers in the Finrisk Study.^**a**^ ^a^ The figure shows the mean plasma LDL cholesterol level by *ANGPTL4* and *ANGPTL8* PTV carrier status with respect to self-reported fasting time. The number of *ANGPTL4* and *ANGPTL8* heterozygotes for each fasting time interval are reported below the fasting time legend. The points indicate means and the error bars 95% confidence intervals. The P values are for the two-sided Welch’s t-test between triglyceride levels of noncarriers and heterozygotes. LDL cholesterol levels of individuals with lipid-lowering therapy were divided by 0.7.
(TIFF)
