# Supplementary material for: ANGPTL8 protein-truncating variant associated with lower serum triglycerides and risk of coronary disease
Source: PLoS Genet. 2021 Apr 28;17(4):e1009501. doi: 10.1371/journal.pgen.1009501 (PMC8109807; doi:10.1371/journal.pgen.1009501)
Supplement: S1 Text — People who contributed to the FinnGen Study are listed in the supplementary text. (PDF) [file pgen.1009501.s001.pdf]

Contributors of FinnGen

Steering Committee

Aarno Palotie Institute for Molecular Medicine Finland, HiLIFE, University of Helsinki, Finland

Mark Daly Institute for Molecular Medicine Finland, HiLIFE, University of Helsinki, Finland

Pharmaceutical companies

Bridget Riley-Gills Abbvie, Chicago, IL, United States

Howard Jacob Abbvie, Chicago, IL, United States

Dirk Paul Astra Zeneca, Cambridge, United Kingdom

Heiko Runz Biogen, Cambridge, MA, United States

Sally John Biogen, Cambridge, MA, United States

Robert Plenge Celgene, Summit, NJ, United States/Bristol Myers Squibb, New York, NY, United States

Mark McCarthy Genentech, San Francisco, CA, United States

Julie Hunkapiller Genentech, San Francisco, CA, United States

Meg Ehm GlaxoSmithKline, Brentford, United Kingdom

Kirsi Auro GlaxoSmithKline, Brentford, United Kingdom

Caroline Fox Merck, Kenilworth, NJ, United States

Anders Mälarstig Pfizer, New York, NY, United States

Katherine Klinger Sanofi, Paris, France

Deepak Raipal Sanofi, Paris, France

Tim Behrens Maze Therapeutics, San Francisco, CA, United States

Robert Yang Janssen Biotech, Beerse, Belgium

Richard Siegel Novartis, Basel, Switzerland

University of Helsinki & Biobanks

Tomi Mäkelä HiLIFE, University of Helsinki, Finland, Finland

Jaakko Kaprio Institute for Molecular Medicine Finland, HiLIFE, Helsinki, Finland, Finland

Petri Virolainen Auria Biobank / University of Turku / Hospital District of Southwest Finland, Turku, Finland

Antti Hakanen Auria Biobank / University of Turku / Hospital District of Southwest Finland, Turku, Finland

Terhi Kilpi	THL Biobank / The National Institute of Health and Welfare Helsinki, Finland
Markus Perola	THL Biobank / The National Institute of Health and Welfare Helsinki, Finland
Jukka Partanen	Finnish Red Cross Blood Service / Finnish Hematology Registry and Clinical Biobank, Helsinki, Finland
Anne Pitkäranta	Helsinki Biobank / Helsinki University and Hospital District of Helsinki and Uusimaa, Helsinki
Juhani Junttila	Northern Finland Biobank Borealis / University of Oulu / Northern Ostrobothnia Hospital District, Oulu, Finland
Raisa Serpi	Northern Finland Biobank Borealis / University of Oulu / Northern Ostrobothnia Hospital District, Oulu, Finland
Tarja Laitinen	Finnish Clinical Biobank Tampere / University of Tampere / Pirkanmaa Hospital District, Tampere, Finland
Johanna Mäkelä	Finnish Clinical Biobank Tampere / University of Tampere / Pirkanmaa Hospital District, Tampere, Finland
Veli-Matti Kosma	Biobank of Eastern Finland / University of Eastern Finland / Northern Savo Hospital District, Kuopio, Finland
Urho Kujala	Central Finland Biobank / University of Jyväskylä / Central Finland Health Care District, Jyväskylä, Finland

Other Experts/ Non-Voting Members

Outi Tuovila	Business Finland, Helsinki, Finland
Raimo Pakkanen	Business Finland, Helsinki, Finland

Scientific Committee

Pharmaceutical companies

Jeffrey Waring	Abbvie, Chicago, IL, United States
Ali Abbasi	Abbvie, Chicago, IL, United States
Mengzhen Liu	Abbvie, Chicago, IL, United States
Ioanna Tachmazidou	Astra Zeneca, Cambridge, United Kingdom
Chia-Yen Chen	Biogen, Cambridge, MA, United States

Heiko Runz Biogen, Cambridge, MA, United States

Shameek Biswas Celgene, Summit, NJ, United States/Bristol Myers Squibb, New York, NY, United States

Julie Hunkapiller Genentech, San Francisco, CA, United States

Meg Ehm GlaxoSmithKline, Brentford, United Kingdom

Neha Raghavan Merck, Kenilworth, NJ, United States

Adriana Huertas-Vazquez Merck, Kenilworth, NJ, United States

Anders Mälarstig Pfizer, New York, NY, United States

Xinli Hu Pfizer, New York, NY, United States

Katherine Klinger Sanofi, Paris, France

Matthias Gossel Sanofi, Paris, France

Robert Graham Maze Therapeutics, San Francisco, CA, United States

Tim Behrens Maze Therapeutics, San Francisco, CA, United States

Beryl Cummings Maze Therapeutics, San Francisco, CA, United States

Wilco Fleuren Janssen Biotech, Beerse, Belgium

Dawn Waterworth Janssen Biotech, Beerse, Belgium

Nicole Renaud Novartis, Basel, Switzerland

Ma´en Obeidat Novartis, Basel, Switzerland

University of Helsinki & Biobanks

Samuli Ripatti Institute for Molecular Medicine Finland, HiLIFE, Helsinki, Finland

Johanna Schleutker Auria Biobank / Univ. of Turku / Hospital District of Southwest Finland, Turku, Finland

Markus Perola THL Biobank / The National Institute of Health and Welfare Helsinki, Finland

Mikko Arvas Finnish Red Cross Blood Service / Finnish Hematology Registry and Clinical Biobank, Helsinki, Finland

Olli Carpén Helsinki Biobank / Helsinki University and Hospital District of Helsinki and Uusimaa, Helsinki

Reetta Hinttala Northern Finland Biobank Borealis / University of Oulu / Northern Ostrobothnia Hospital District, Oulu, Finland

Johannes Kettunen Northern Finland Biobank Borealis / University of Oulu / Northern Ostrobothnia Hospital District, Oulu, Finland

Johanna Mäkelä Finnish Clinical Biobank Tampere / University of Tampere / Pirkanmaa Hospital District, Tampere, Finland

Arto Mannermaa Biobank of Eastern Finland / University of Eastern Finland / Northern Savo Hospital District, Kuopio, Finland

Jari Laukkanen Central Finland Biobank / University of Jyväskylä / Central Finland Health Care District, Jyväskylä, Finland

Urho Kujala Central Finland Biobank / University of Jyväskylä / Central Finland Health Care District, Jyväskylä, Finland

Clinical Groups

Neurology Group

Reetta Kälviäinen	Northern Savo Hospital District, Kuopio, Finland
Valtteri Julkunen	Northern Savo Hospital District, Kuopio, Finland
Hilkka Soininen	Northern Savo Hospital District, Kuopio, Finland
Anne Remes	Northern Ostrobothnia Hospital District, Oulu, Finland
Mikko Hiltunen	Northern Savo Hospital District, Kuopio, Finland
Jukka Peltola	Pirkanmaa Hospital District, Tampere, Finland
Pentti Tienari	Hospital District of Helsinki and Uusimaa, Helsinki, Finland
Juha Rinne	Hospital District of Southwest Finland, Turku, Finland
Roosa Kallionpää	Hospital District of Southwest Finland, Turku, Finland
Ali Abbasi	Abbvie, Chicago, IL, United States
Adam Ziemann	Abbvie, Chicago, IL, United States
Jeffrey Waring	Abbvie, Chicago, IL, United States
Sahar Esmaeeli	Abbvie, Chicago, IL, United States
Nizar Smaoui	Abbvie, Chicago, IL, United States
Anne Lehtonen	Abbvie, Chicago, IL, United States
Susan Eaton	Biogen, Cambridge, MA, United States
Heiko Runz	Biogen, Cambridge, MA, United States
Sanni Lahdenperä	Biogen, Cambridge, MA, United States
Janet van Adelsberg	Celgene, Summit, NJ, United States/ Bristol Myers Squibb, New York, NY, United States

Shameek Biswas	Celgene, Summit, NJ, United States/ Bristol Myers Squibb, New York, NY, United States
Julie Hunkapiller	Genentech, San Francisco, CA, United States
Natalie Bowers	Genentech, San Francisco, CA, United States
Edmond Teng	Genentech, San Francisco, CA, United States
Sarah Pendergrass	Genentech, San Francisco, CA, United States
Onuralp Soylemez	Merck, Kenilworth, NJ, United States
Kari Linden	Pfizer, New York, NY, United States
Fanli Xu	GlaxoSmithKline, Brentford, United Kingdom
David Pulford	GlaxoSmithKline, Brentford, United Kingdom
Kirsi Auro	GlaxoSmithKline, Brentford, United Kingdom
Laura Addis	GlaxoSmithKline, Brentford, United Kingdom
John Eicher	GlaxoSmithKline, Brentford, United Kingdom
Minna Raivio	Hospital District of Helsinki and Uusimaa, Helsinki, Finland
Sarah Pendergrass	Genentech, San Francisco, CA, United States
Beryl Cummings	Maze Therapeutics, San Francisco, CA, United States
Juulia Partanen	Institute for Molecular Medicine Finland, HiLIFE, University of Helsinki, Finland

Gastroenterology Group

Martti Färkkilä	Hospital District of Helsinki and Uusimaa, Helsinki, Finland
Jukka Koskela	Hospital District of Helsinki and Uusimaa, Helsinki, Finland
Sampsa Pikkarainen	Hospital District of Helsinki and Uusimaa, Helsinki, Finland
Airi Jussila	Pirkanmaa Hospital District, Tampere, Finland
Katri Kaukinen	Pirkanmaa Hospital District, Tampere, Finland
Timo Blomster	Northern Ostrobothnia Hospital District, Oulu, Finland
Mikko Kiviniemi	Northern Savo Hospital District, Kuopio, Finland
Markku Voutilainen	Hospital District of Southwest Finland, Turku, Finland
Ali Abbasi	Abbvie, Chicago, IL, United States
Graham Heap	Abbvie, Chicago, IL, United States
Jeffrey Waring	Abbvie, Chicago, IL, United States
Nizar Smaoui	Abbvie, Chicago, IL, United States

Fedik Rahimov	Abbvie, Chicago, IL, United States
Anne Lehtonen	Abbvie, Chicago, IL, United States
Keith Usiskin	Celgene, Summit, NJ, United States/ Bristol Myers Squibb, New York, NY, United States
Tim Lu	Genentech, San Francisco, CA, United States
Natalie Bowers	Genentech, San Francisco, CA, United States
Danny Oh	Genentech, San Francisco, CA, United States
Sarah Pendergrass	Genentech, San Francisco, CA, United States
Kirsi Kalpala	Pfizer, New York, NY, United States
Melissa Miller	Pfizer, New York, NY, United States
Xinli Hu	Pfizer, New York, NY, United States
Linda McCarthy	GlaxoSmithKline, Brentford, United Kingdom
Onuralp Soylemez	Merck, Kenilworth, NJ, United States
Mark Daly	Institute for Molecular Medicine Finland, HiLIFE, University of Helsinki, Finland

Rheumatology Group

Kari Eklund	Hospital District of Helsinki and Uusimaa, Helsinki, Finland
Antti Palomäki	Hospital District of Southwest Finland, Turku, Finland
Pia Isomäki	Pirkanmaa Hospital District, Tampere, Finland
Laura Pirilä	Hospital District of Southwest Finland, Turku, Finland
Oili Kaipiainen-Seppänen	Northern Savo Hospital District, Kuopio, Finland
Johanna Huhtakangas	Northern Ostrobothnia Hospital District, Oulu, Finland
Ali Abbasi	Abbvie, Chicago, IL, United States
Jeffrey Waring	Abbvie, Chicago, IL, United States
Fedik Rahimov	Abbvie, Chicago, IL, United States
Apinya Lertratanakul	Abbvie, Chicago, IL, United States
Nizar Smaoui	Abbvie, Chicago, IL, United States
Anne Lehtonen	Abbvie, Chicago, IL, United States
David Close	Astra Zeneca, Cambridge, United Kingdom
Marla Hochfeld	Celgene, Summit, NJ, United States/ Bristol Myers Squibb, New York, NY, United States
Natalie Bowers	Genentech, San Francisco, CA, United States

Sarah Pendergrass	Genentech, San Francisco, CA, United States
Onuralp Soylemez	Merck, Kenilworth, NJ, United States
Kirsi Kalpala	Pfizer, New York, NY, United States
Nan Bing	Pfizer, New York, NY, United States
Xinli Hu	Pfizer, New York, NY, United States
Jorge Esparza Gordillo	GlaxoSmithKline, Brentford, United Kingdom
Kirsi Auro	GlaxoSmithKline, Brentford, United Kingdom
Dawn Waterworth	Janssen Biotech, Beerse, Belgium
Nina Mars	Institute for Molecular Medicine Finland, HiLIFE, Helsinki, Finland

Pulmonology Group

Tarja Laitinen	Pirkanmaa Hospital District, Tampere, Finland
Margit Pelkonen	Northern Savo Hospital District, Kuopio, Finland
Paula Kauppi	Hospital District of Helsinki and Uusimaa, Helsinki, Finland
Hannu Kankaanranta	Pirkanmaa Hospital District, Tampere, Finland
Terttu Harju	Northern Ostrobothnia Hospital District, Oulu, Finland
Riitta Lahesmaa	Hospital District of Southwest Finland, Turku, Finland
Nizar Smaoui	Abbvie, Chicago, IL, United States
Alex Mackay	Astra Zeneca, Cambridge, United Kingdom
Glenda Lassi	Astra Zeneca, Cambridge, United Kingdom
Susan Eaton	Biogen, Cambridge, MA, United States
Steven Greenberg	Celgene, Summit, NJ, United States/ Bristol Myers Squibb, New York, NY, United States
Hubert Chen	Genentech, San Francisco, CA, United States
Sarah Pendergrass	Genentech, San Francisco, CA, United States
Natalie Bowers	Genentech, San Francisco, CA, United States
Joanna Betts	GlaxoSmithKline, Brentford, United Kingdom
Soumitra Ghosh	GlaxoSmithKline, Brentford, United Kingdom
Kirsi Auro	GlaxoSmithKline, Brentford, United Kingdom
Rajashree Mishra	GlaxoSmithKline, Brentford, United Kingdom
Sina Rüeger	Institute for Molecular Medicine Finland, HiLIFE, University of Helsinki, Finland

Cardiometabolic Diseases Group

Teemu Niiranen The National Institute of Health and Welfare Helsinki, Finland

Felix Vaura The National Institute of Health and Welfare Helsinki, Finland

Veikko Salomaa The National Institute of Health and Welfare Helsinki, Finland

Markus Juonala Hospital District of Southwest Finland, Turku, Finland

Kaj Metsärinne Hospital District of Southwest Finland, Turku, Finland

Mika Kähönen Pirkanmaa Hospital District, Tampere, Finland

Juhani Junttila Northern Ostrobothnia Hospital District, Oulu, Finland

Markku Laakso Northern Savo Hospital District, Kuopio, Finland

Jussi Pihlajamäki Northern Savo Hospital District, Kuopio, Finland

Daniel Gordin Hospital District of Helsinki and Uusimaa, Helsinki, Finland

Juha Sinisalo Hospital District of Helsinki and Uusimaa, Helsinki, Finland

Marja-Riitta Taskinen Hospital District of Helsinki and Uusimaa, Helsinki, Finland

Tiinamaija Tuomi Hospital District of Helsinki and Uusimaa, Helsinki, Finland

Jari Laukkanen Central Finland Health Care District, Jyväskylä, Finland

Benjamin Challis Astra Zeneca, Cambridge, United Kingdom

Dirk Paul Astra Zeneca, Cambridge, United Kingdom

Julie Hunkapiller Genentech, San Francisco, CA, United States

Natalie Bowers Genentech, San Francisco, CA, United States

Sarah Pendergrass Genentech, San Francisco, CA, United States

Onuralp Soylemez Merck, Kenilworth, NJ, United States

Jaakko Parkkinen Pfizer, New York, NY, United States

Melissa Miller Pfizer, New York, NY, United States

Russell Miller Pfizer, New York, NY, United States

Audrey Chu GlaxoSmithKline, Brentford, United Kingdom

Kirsi Auro GlaxoSmithKline, Brentford, United Kingdom

Keith Usiskin Celgene, Summit, NJ, United States/ Bristol Myers Squibb, New York, NY, United States

Amanda Elliott Institute for Molecular Medicine Finland, HiLIFE, University of Helsinki, Finland / Broad Institute, Cambridge, MA, United States

Joel Rämö	Institute for Molecular Medicine Finland, HiLIFE, University of Helsinki, Finland
Samuli Ripatti	Institute for Molecular Medicine Finland, HiLIFE, University of Helsinki, Finland
Mary Pat Reeve	Institute for Molecular Medicine Finland, HiLIFE, University of Helsinki, Finland
Sanni Ruotsalainen	Institute for Molecular Medicine Finland, HiLIFE, University of Helsinki, Finland

Oncology Group

Tuomo Meretoja	Hospital District of Helsinki and Uusimaa, Helsinki, Finland
Heikki Joensuu	Hospital District of Helsinki and Uusimaa, Helsinki, Finland
Olli Carpén	Hospital District of Helsinki and Uusimaa, Helsinki, Finland
Lauri Aaltonen	Hospital District of Helsinki and Uusimaa, Helsinki, Finland
Johanna Mattson	Hospital District of Helsinki and Uusimaa, Helsinki, Finland
Annika Auranen	Pirkanmaa Hospital District , Tampere, Finland
Peeter Karihtala	Northern Ostrobothnia Hospital District, Oulu, Finland
Saila Kauppila	Northern Ostrobothnia Hospital District, Oulu, Finland
Päivi Auvinen	Northern Savo Hospital District, Kuopio, Finland
Klaus Elenius	Hospital District of Southwest Finland, Turku, Finland
Johanna Schleutker	Hospital District of Southwest Finland, Turku, Finland
Relja Popovic	Abbvie, Chicago, IL, United States
Jeffrey Waring	Abbvie, Chicago, IL, United States
Bridget Riley-Gillis	Abbvie, Chicago, IL, United States
Anne Lehtonen	Abbvie, Chicago, IL, United States
Jennifer Schutzman	Genentech, San Francisco, CA, United States
Julie Hunkapiller	Genentech, San Francisco, CA, United States
Natalie Bowers	Genentech, San Francisco, CA, United States
Sarah Pendergrass	Genentech, San Francisco, CA, United States
Andrey Loboda	Merck, Kenilworth, NJ, United States
Aparna Chhibber	Merck, Kenilworth, NJ, United States
Heli Lehtonen	Pfizer, New York, NY, United States
Stefan McDonough	Pfizer, New York, NY, United States
Marika Crohns	Sanofi, Paris, France

Sauli Vuoti Sanofi, Paris, France

Diptee Kulkarni GlaxoSmithKline, Brentford, United Kingdom

Kirsi Auro GlaxoSmithKline, Brentford, United Kingdom

Esa Pitkänen Institute for Molecular Medicine Finland, HiLIFE, University of Helsinki, Finland

Nina Mars Institute for Molecular Medicine Finland, HiLIFE, University of Helsinki, Finland

Mark Daly Institute for Molecular Medicine Finland, HiLIFE, University of Helsinki, Finland

Opthalmology Group

Kai Kaarniranta Northern Savo Hospital District, Kuopio, Finland

Joni A Turunen Hospital District of Helsinki and Uusimaa, Helsinki, Finland

Terhi Ollila Hospital District of Helsinki and Uusimaa, Helsinki, Finland

Sanna Seitsonen Hospital District of Helsinki and Uusimaa, Helsinki, Finland

Hannu Uusitalo Pirkanmaa Hospital District, Tampere, Finland

Vesa Aaltonen Hospital District of Southwest Finland, Turku, Finland

Hannele Uusitalo-Järvinen Pirkanmaa Hospital District, Tampere, Finland

Marja Luodonpää Northern Ostrobothnia Hospital District, Oulu, Finland

Nina Hautala Northern Ostrobothnia Hospital District, Oulu, Finland

Mengzhen Liu Abbvie, Chicago, IL, United States

Heiko Runz Biogen, Cambridge, MA, United States

Stephanie Loomis Biogen, Cambridge, MA, United States

Erich Strauss Genentech, San Francisco, CA, United States

Natalie Bowers Genentech, San Francisco, CA, United States

Hao Chen Genentech, San Francisco, CA, United States

Sarah Pendergrass Genentech, San Francisco, CA, United States

Anna Podgornaia Merck, Kenilworth, NJ, United States

Juha Karjalainen Institute for Molecular Medicine Finland, HiLIFE, University of Helsinki, Finland / Broad Institute, Cambridge, MA, United States

Esa Pitkänen Institute for Molecular Medicine Finland, HiLIFE, University of Helsinki, Finland

Dermatology Group

Kaisa Tasanen	Northern Ostrobothnia Hospital District, Oulu, Finland
Laura Huilaja	Northern Ostrobothnia Hospital District, Oulu, Finland
Katariina Hannula-Jouppi	Hospital District of Helsinki and Uusimaa, Helsinki, Finland
Teea Salmi	Pirkanmaa Hospital District, Tampere, Finland
Sirkku Peltonen	Hospital District of Southwest Finland, Turku, Finland
Leena Koulu	Hospital District of Southwest Finland, Turku, Finland
Kirsi Kalpala	Pfizer, New York, NY, United States
Ying Wu	Pfizer, New York, NY, United States
David Choy	Genentech, San Francisco, CA, United States
Sarah Pendergrass	Genentech, San Francisco, CA, United States
Nizar Smaoui	Abbvie, Chicago, IL, United States
Fedik Rahimov	Abbvie, Chicago, IL, United States
Anne Lehtonen	Abbvie, Chicago, IL, United States
Dawn Waterworth	Janssen Biotech, Beerse, Belgium

Odontology Group

Pirkko Pussinen	Hospital District of Helsinki and Uusimaa, Helsinki, Finland
Aino Salminen	Hospital District of Helsinki and Uusimaa, Helsinki, Finland
Tuula Salo	Hospital District of Helsinki and Uusimaa, Helsinki, Finland
David Rice	Hospital District of Helsinki and Uusimaa, Helsinki, Finland
Pekka Nieminen	Hospital District of Helsinki and Uusimaa, Helsinki, Finland
Ulla Palotie	Hospital District of Helsinki and Uusimaa, Helsinki, Finland
Juha Sinisalo	Hospital District of Helsinki and Uusimaa, Helsinki, Finland
Maria Siponen	Northern Savo Hospital District, Kuopio, Finland
Liisa Suominen	Northern Savo Hospital District, Kuopio, Finland
Päivi Mäntylä	Northern Savo Hospital District, Kuopio, Finland
Ulvi Gursoy	Hospital District of Southwest Finland, Turku, Finland
Vuokko Anttonen	Northern Ostrobothnia Hospital District, Oulu, Finland
Kirsi Sipilä	Northern Ostrobothnia Hospital District, Oulu, Finland
Sarah Pendergrass	Genentech, San Francisco, CA, United States

Women's Health and Reproduction Group

Hannele Laivuori	Institute for Molecular Medicine Finland, HiLIFE, University of Helsinki, Finland
Venla Kurra	Pirkanmaa Hospital District, Tampere, Finland
Oskari Heikinheimo	Hospital District of Helsinki and Uusimaa, Helsinki, Finland
Ilkka Kalliala	Hospital District of Helsinki and Uusimaa, Helsinki, Finland
Laura Kotaniemi-Talonen	Pirkanmaa Hospital District, Tampere, Finland
Kari Nieminen	Pirkanmaa Hospital District, Tampere, Finland
Päivi Polo	Hospital District of Southwest Finland, Turku, Finland
Kaarin Mäkikallio	Hospital District of Southwest Finland, Turku, Finland
Eeva Ekholm	Hospital District of Southwest Finland, Turku, Finland
Marja Vääräsmäki	Northern Ostrobothnia Hospital District, Oulu, Finland
Outi Uimari	Northern Ostrobothnia Hospital District, Oulu, Finland
Laure Morin-Papunen	Northern Ostrobothnia Hospital District, Oulu, Finland
Marjo Tuppurainen	Northern Savo Hospital District, Kuopio, Finland
Katja Kivinen	Institute for Molecular Medicine Finland, HiLIFE, University of Helsinki, Finland
Elisabeth Widen	Institute for Molecular Medicine Finland, HiLIFE, University of Helsinki, Finland
Taru Tukiainen	Institute for Molecular Medicine Finland, HiLIFE, University of Helsinki, Finland
Mary Pat Reeve	Institute for Molecular Medicine Finland, HiLIFE, University of Helsinki, Finland
Mark Daly	Institute for Molecular Medicine Finland, HiLIFE, University of Helsinki, Finland
Liu Aoxing	Institute for Molecular Medicine Finland, HiLIFE, University of Helsinki, Finland
Eija Laakkonen	University of Jyväskylä, Jyväskylä, Finland
Niko Välimäki	University of Helsinki, Helsinki, Finland
Lauri Aaltonen	Hospital District of Helsinki and Uusimaa, Helsinki, Finland
Johannes Kettunen	Northern Ostrobothnia Hospital District, Oulu, Finland
Mikko Arvas	Finnish Red Cross Blood Service, Helsinki, Finland
Jeffrey Waring	Abbvie, Chicago, IL, United States
Bridget Riley-Gillis	Abbvie, Chicago, IL, United States
Mengzhen Liu	Abbvie, Chicago, IL, United States
Janet Kumar	GlaxoSmithKline, Brentford, United Kingdom

Kirsi Auro	GlaxoSmithKline, Brentford, United Kingdom
Andrea Ganna	Institute for Molecular Medicine Finland, HiLIFE, University of Helsinki, Finland
Sarah Pendergrass	Genentech, San Francisco, CA, United States

FinnGen Analysis working group

Justin Wade Davis	Abbvie, Chicago, IL, United States
Bridget Riley-Gillis	Abbvie, Chicago, IL, United States
Danjuma Quarless	Abbvie, Chicago, IL, United States
Fedik Rahimov	Abbvie, Chicago, IL, United States
Sahar Esmaeeli	Abbvie, Chicago, IL, United States
Slavé Petrovski	Astra Zeneca, Cambridge, United Kingdom
Eleonor Wigmore	Astra Zeneca, Cambridge, United Kingdom
Adele Mitchell	Biogen, Cambridge, MA, United States
Benjamin Sun	Biogen, Cambridge, MA, United States
Ellen Tsai	Biogen, Cambridge, MA, United States
Denis Baird	Biogen, Cambridge, MA, United States
Paola Bronson	Biogen, Cambridge, MA, United States
Ruoyu Tian	Biogen, Cambridge, MA, United States
Stephanie Loomis	Biogen, Cambridge, MA, United States
Yunfeng Huang	Biogen, Cambridge, MA, United States
Joseph Maranville	Celgene, Summit, NJ, United States/ Bristol Myers Squibb, New York, NY, United States
Shameek Biswas	Celgene, Summit, NJ, United States/ Bristol Myers Squibb, New York, NY, United States
Elmutaz Mohammed	Celgene, Summit, NJ, United States/ Bristol Myers Squibb, New York, NY, United States
Samir Wadhawan	Celgene, Summit, NJ, United States/ Bristol Myers Squibb, New York, NY, United States
Erika Kvikstad	Celgene, Summit, NJ, United States/ Bristol Myers Squibb, New York, NY, United States
Minal Caliskan	Celgene, Summit, NJ, United States/ Bristol Myers Squibb, New York, NY, United States
Diana Chang	Genentech, San Francisco, CA, United States
Julie Hunkapiller	Genentech, San Francisco, CA, United States
Tushar Bhangale	Genentech, San Francisco, CA, United States
Natalie Bowers	Genentech, San Francisco, CA, United States
Sarah Pendergrass	Genentech, San Francisco, CA, United States

Kirill Shkura Merck, Kenilworth, NJ, United States

Victor Neduva Merck, Kenilworth, NJ, United States

Xing Chen Pfizer, New York, NY, United States

Åsa Hedman Pfizer, New York, NY, United States

Karen S King GlaxoSmithKline, Brentford, United Kingdom

Padhraig Gormley GlaxoSmithKline, Brentford, United Kingdom

Jimmy Liu GlaxoSmithKline, Brentford, United Kingdom

Clarence Wang Sanofi, Paris, France

Ethan Xu Sanofi, Paris, France

Franck Auge Sanofi, Paris, France

Clement Chatelain Sanofi, Paris, France

Deepak Rajpal Sanofi, Paris, France

Dongyu Liu Sanofi, Paris, France

Katherine Call Sanofi, Paris, France

Tai-He Xia Sanofi, Paris, France

Beryl Cummings Maze Therapeutics, San Francisco, CA, United States

Matt Brauer Maze Therapeutics, San Francisco, CA, United States

Huilei Xu Novartis, Basel, Switzerland

Amy Cole Novartis, Basel, Switzerland

Jonathan Chung Novartis, Basel, Switzerland

Jaison Jacob Novartis, Basel, Switzerland

Katrina de Lange Novartis, Basel, Switzerland

Jonas Zierer Novartis, Basel, Switzerland

Mitja Kurki Institute for Molecular Medicine Finland, HiLIFE, University of Helsinki, Finland / Broad Institute, Cambridge, MA, United States

Samuli Ripatti Institute for Molecular Medicine Finland, HiLIFE, University of Helsinki, Finland

Mark Daly Institute for Molecular Medicine Finland, HiLIFE, University of Helsinki, Finland

Juha Karjalainen Institute for Molecular Medicine Finland, HiLIFE, University of Helsinki, Finland / Broad Institute, Cambridge, MA, United States

Aki Havulinna Institute for Molecular Medicine Finland, HiLIFE, University of Helsinki, Finland

Juha Mehtonen	Institute for Molecular Medicine Finland, HiLIFE, University of Helsinki, Finland
Priit Palta	Institute for Molecular Medicine Finland, HiLIFE, University of Helsinki, Finland
Shabbeer Hassan	Institute for Molecular Medicine Finland, HiLIFE, University of Helsinki, Finland
Pietro Della Briotta Parolo	Institute for Molecular Medicine Finland, HiLIFE, University of Helsinki, Finland
Wei Zhou	Broad Institute, Cambridge, MA, United States
Mutaamba Maasha	Broad Institute, Cambridge, MA, United States
Shabbeer Hassan	Institute for Molecular Medicine Finland, HiLIFE, University of Helsinki, Finland
Susanna Lemmelä	Institute for Molecular Medicine Finland, HiLIFE, University of Helsinki, Finland
Manuel Rivas	University of Stanford, Stanford, CA, United States
Aarno Palotie	Institute for Molecular Medicine Finland, HiLIFE, University of Helsinki, Finland
Arto Lehisto	Institute for Molecular Medicine Finland, HiLIFE, University of Helsinki, Finland
Andrea Ganna	Institute for Molecular Medicine Finland, HiLIFE, University of Helsinki, Finland
Vincent Llorens	Institute for Molecular Medicine Finland, HiLIFE, University of Helsinki, Finland
Hannele Laivuori	Institute for Molecular Medicine Finland, HiLIFE, University of Helsinki, Finland
Mari E Niemi	Institute for Molecular Medicine Finland, HiLIFE, University of Helsinki, Finland
Taru Tukiainen	Institute for Molecular Medicine Finland, HiLIFE, University of Helsinki, Finland
Mary Pat Reeve	Institute for Molecular Medicine Finland, HiLIFE, University of Helsinki, Finland
Henrike Heyne	Institute for Molecular Medicine Finland, HiLIFE, University of Helsinki, Finland
Nina Mars	Institute for Molecular Medicine Finland, HiLIFE, University of Helsinki, Finland
Kimmo Palin	University of Helsinki, Helsinki, Finland
Javier Garcia-Tabuenca	University of Tampere, Tampere, Finland
Harri Siirtola	University of Tampere, Tampere, Finland
Tuomo Kiiskinen	Institute for Molecular Medicine Finland, HiLIFE, University of Helsinki, Finland
Jiwoo Lee	Institute for Molecular Medicine Finland, HiLIFE, University of Helsinki, Finland / Broad Institute, Cambridge, MA, United States
Kristin Tsuo	Institute for Molecular Medicine Finland, HiLIFE, University of Helsinki, Finland / Broad Institute, Cambridge, MA, United States
Amanda Elliott	Institute for Molecular Medicine Finland, HiLIFE, University of Helsinki, Finland / Broad Institute, Cambridge, MA, United States
Kati Kristiansson	THL Biobank / The National Institute of Health and Welfare Helsinki, Finland

Mikko Arvas Finnish Red Cross Blood Service / Finnish Hematology Registry and Clinical Biobank, Helsinki, Finland

Kati Hyvärinen Finnish Red Cross Blood Service, Helsinki, Finland

Jarmo Ritari Finnish Red Cross Blood Service, Helsinki, Finland

Miika Koskinen Helsinki Biobank / Helsinki University and Hospital District of Helsinki and Uusimaa, Helsinki

Olli Carpén Helsinki Biobank / Helsinki University and Hospital District of Helsinki and Uusimaa, Helsinki

Johannes Kettunen Northern Finland Biobank Borealis / University of Oulu / Northern Ostrobothnia Hospital District, Oulu, Finland

Katri Pylkäs University of Oulu, Oulu, Finland

Marita Kalaoja University of Oulu, Oulu, Finland

Minna Karjalainen University of Oulu, Oulu, Finland

Tuomo Mantere Northern Finland Biobank Borealis / University of Oulu / Northern Ostrobothnia Hospital District, Oulu, Finland

Eeva Kangasniemi Finnish Clinical Biobank Tampere / University of Tampere / Pirkanmaa Hospital District, Tampere, Finland

Sami Heikkinen University of Eastern Finland, Kuopio, Finland

Arto Mannermaa Biobank of Eastern Finland / University of Eastern Finland / Northern Savo Hospital District, Kuopio, Finland

Eija Laakkonen University of Jyväskylä, Jyväskylä, Finland

Samuel Heron University of Turku, Turku, Finland

Dhanaprakash Jambulingam University of Turku, Turku, Finland

Venkat Subramaniam Rathinakannan University of Turku, Turku, Finland

Nina Pitkänen Auria Biobank / University of Turku / Hospital District of Southwest Finland, Turku, Finland

Biobank directors

Lila Kallio Auria Biobank / University of Turku / Hospital District of Southwest Finland, Turku, Finland

Sirpa Soini THL Biobank / The National Institute of Health and Welfare Helsinki, Finland

Jukka Partanen Finnish Red Cross Blood Service / Finnish Hematology Registry and Clinical Biobank, Helsinki, Finland

Eero Punkka Helsinki Biobank / Helsinki University and Hospital District of Helsinki and Uusimaa, Helsinki

Raisa Serpi Northern Finland Biobank Borealis / University of Oulu / Northern Ostrobothnia Hospital District, Oulu, Finland

Johanna Mäkelä Finnish Clinical Biobank Tampere / University of Tampere / Pirkanmaa Hospital District, Tampere, Finland

Veli-Matti Kosma Biobank of Eastern Finland / University of Eastern Finland / Northern Savo Hospital District, Kuopio, Finland

Teijo Kuopio Central Finland Biobank / University of Jyväskylä / Central Finland Health Care District, Jyväskylä, Finland

FinnGen Teams

Administration

Anu Jalanko Institute for Molecular Medicine Finland, HiLIFE, University of Helsinki, Finland

Huei-Yi Shen Institute for Molecular Medicine Finland, HiLIFE, University of Helsinki, Finland

Risto Kajanne Institute for Molecular Medicine Finland, HiLIFE, University of Helsinki, Finland

Mervi Aavikko Institute for Molecular Medicine Finland, HiLIFE, University of Helsinki, Finland

Analysis

Mitja Kurki Institute for Molecular Medicine Finland, HiLIFE, University of Helsinki, Finland / Broad Institute, Cambridge, MA, United States

Juha Karjalainen Institute for Molecular Medicine Finland, HiLIFE, University of Helsinki, Finland / Broad Institute, Cambridge, MA, United States

Pietro Della Briotta Parolo Institute for Molecular Medicine Finland, HiLIFE, University of Helsinki, Finland

Arto Lehisto Institute for Molecular Medicine Finland, HiLIFE, University of Helsinki, Finland

Juha Mehtonen Institute for Molecular Medicine Finland, HiLIFE, University of Helsinki, Finland

Wei Zhou	Broad Institute, Cambridge, MA, United States
Masahiro Kanai	Broad Institute, Cambridge, MA, United States
Mutaamba Maasha	Broad Institute, Cambridge, MA, United States

Clinical Endpoint Development

Hannele Laivuori	Institute for Molecular Medicine Finland, HiLIFE, University of Helsinki, Finland
Aki Havulinna	Institute for Molecular Medicine Finland, HiLIFE, University of Helsinki, Finland
Susanna Lemmelä	Institute for Molecular Medicine Finland, HiLIFE, University of Helsinki, Finland
Tuomo Kiiskinen	Institute for Molecular Medicine Finland, HiLIFE, University of Helsinki, Finland
L. Elisa Lahtela	Institute for Molecular Medicine Finland, HiLIFE, University of Helsinki, Finland
Matti Peura	Institute for Molecular Medicine Finland, HiLIFE, University of Helsinki, Finland

Communication

Mari Kaunisto	Institute for Molecular Medicine Finland, HiLIFE, University of Helsinki, Finland

Data Management and IT Infrastructure

Elina Kilpeläinen	Institute for Molecular Medicine Finland, HiLIFE, University of Helsinki, Finland
Timo P. Sipilä	Institute for Molecular Medicine Finland, HiLIFE, University of Helsinki, Finland
Georg Brein	Institute for Molecular Medicine Finland, HiLIFE, University of Helsinki, Finland
Oluwaseun A. Dada	Institute for Molecular Medicine Finland, HiLIFE, University of Helsinki, Finland
Awaisa Ghazal	Institute for Molecular Medicine Finland, HiLIFE, University of Helsinki, Finland
Anastasia Shcherban	Institute for Molecular Medicine Finland, HiLIFE, University of Helsinki, Finland

Genotyping

Kati Donner	Institute for Molecular Medicine Finland, HiLIFE, University of Helsinki, Finland
Timo P. Sipilä	Institute for Molecular Medicine Finland, HiLIFE, University of Helsinki, Finland

Sample Collection Coordination

Anu Loukola Helsinki Biobank / Helsinki University and Hospital District of Helsinki and Uusimaa, Helsinki

Sample Logistics

Päivi Laiho THL Biobank / The National Institute of Health and Welfare Helsinki, Finland

Tuuli Sistonen THL Biobank / The National Institute of Health and Welfare Helsinki, Finland

Essi Kaiharju THL Biobank / The National Institute of Health and Welfare Helsinki, Finland

Markku Laukkanen THL Biobank / The National Institute of Health and Welfare Helsinki, Finland

Elina Järvensivu THL Biobank / The National Institute of Health and Welfare Helsinki, Finland

Sini Lähteenmäki THL Biobank / The National Institute of Health and Welfare Helsinki, Finland

Lotta Männikkö THL Biobank / The National Institute of Health and Welfare Helsinki, Finland

Regis Wong THL Biobank / The National Institute of Health and Welfare Helsinki, Finland

Registry Data Operations

Hannele Mattsson THL Biobank / The National Institute of Health and Welfare Helsinki, Finland

Kati Kristiansson THL Biobank / The National Institute of Health and Welfare Helsinki, Finland

Susanna Lemmelä Institute for Molecular Medicine Finland, HiLIFE, University of Helsinki, Finland

Sami Koskelainen THL Biobank / The National Institute of Health and Welfare Helsinki, Finland

Tero Hiekkalinna THL Biobank / The National Institute of Health and Welfare Helsinki, Finland

Teemu Paajanen THL Biobank / The National Institute of Health and Welfare Helsinki, Finland

Sequencing Informatics

Priit Palta Institute for Molecular Medicine Finland, HiLIFE, University of Helsinki, Finland

Kalle Pärn Institute for Molecular Medicine Finland, HiLIFE, University of Helsinki, Finland

Shuang Luo Institute for Molecular Medicine Finland, HiLIFE, University of Helsinki, Finland

Vishal Sinha Institute for Molecular Medicine Finland, HiLIFE, University of Helsinki, Finland

Trajectory Team

Tarja Laitinen Pirkanmaa Hospital District, Tampere, Finland

Harri Siirtola University of Tampere, Tampere, Finland

Javier Gracia-Tabuenca University of Tampere, Tampere, Finland

Mika Helminen University of Tampere, Tampere, Finland

Tiina Luukkaala University of Tampere, Tampere, Finland

Iida Vähätalo University of Tampere, Tampere, Finland

Data protection officer

Tero Jyrhämä Institute for Molecular Medicine Finland, HiLIFE, University of Helsinki, Finland

FinBB - Finnish biobank cooperative

Marco Hautalahti

Laura Mustaniemi

Mirkka Koivusalo

Sarah Smith

Tom Southerington
