# Supplementary material for: ANGPTL8 protein-truncating variant associated with lower serum triglycerides and risk of coronary disease
Source: PLoS Genet. 2021 Apr 28;17(4):e1009501. doi: 10.1371/journal.pgen.1009501 (PMC8109807; doi:10.1371/journal.pgen.1009501)
Supplement: S2 Text — A detailed description of the measures used in genotyping, quality control and statistical analyses of the Finrisk and FinnGen Study samples is included in this document. (DOCX) [file pgen.1009501.s002.docx]

# ****Supporting information****

*ANGPTL8* protein-truncating variant associated with lower serum triglycerides and risk of coronary disease

## Table of contents

Supplementary methods 2

1. Genotyping 2
2. Sample and variant level quality control 2
3. Statistical analysis 4
4. References 5

## Supplementary methods

### ****Genotyping****

27,294 Finrisk Study[1] samples were genotyped using the following arrays: the HumanCoreExome BeadChip, the Human610-Quad BeadChip, the Affymetrix6.0, and the Infinium HumanOmniExpress (Illumina Inc., San Diego, CA, USA). The genotype calling of variants on Human610-Quad BeadChip and HumanOmniExpress were performed with Illuminus and the variants on the HumanCoreExome chip with GenomeStudio and ZCall[2] (www.github.com/jigold/zCall) at the Institute for Molecular Medicine Finland (FIMM).

194,181 FinnGen samples were genotyped using the custom AxiomGT1 Affymetrix array or various Illumina arrays. The individuals not genotyped on the AxiomGT1 Affymetrix array came from 29 merged datasets from the Finrisk, Botnia, H2000/2011, Generisk and Psychiatric Family Collections, Auria, Borealis, DIME, FT17, HBP, Iddmgen, VPU and YA cohorts (Table S1).

#### Genotype calls and imputation

The genotype calling of Human610-Quad BeadChip and Infinium HumanOmniExpress variants were performed with Illuminus, version 2 (www.github.com/wtsi-npg/Illuminus/blob/master/illuminus.cc), and the variants on HumanCoreExome BeadChip with GenomeStudio Software, version 2011.1, and ZCall.

After array genotyping the Finrisk samples’ haplotype phases were estimated using Eagle2[3], version 2.3.5 (data.broadinstitute.org/alkesgroup/Eagle/). Genotype imputation was carried out using IMPUTE2[4], version 2.3.2 (mathgen.stats.ox.ac.uk/impute/impute_v2.html), and a Finnish ancestry-specific reference panel consisting of 2,690 deep-coverage (25-30x) whole-genome sequences and 5,092 whole-exome sequences. With the -merge_ref_panels option in IMPUTE2 we were able to combine the whole-genome and whole-exome sequences into one genotype imputation reference panel. Imputed variants were filtered with the IMPUTE2 genotype information score > 0.7.

The estimation of haplotype phases of FinnGen samples were carried out with Eagle2. The genotype imputation in the FinnGen data was carried out using Beagle[5], version 4.1 (faculty.washington.edu/browning/beagle/beagle.html), and a Finnish ancestry-specific reference panel consisting of 3,775 deep-coverage (25-30x) whole-genome sequences. The four lipid-associated PTVs were directly genotyped on the AxiomGT1 Affymetrix array (www.finngen.fi/en/researchers/genotyping) and the average IMPUTE2 genotype information score metric exceeded 0.93 for all four variants. The number of samples before and after genotype imputation, as well as the number of chip genotyped variants for each genotyping batch are listed in S1 Table. The full genotyping and imputation protocol for FinnGen data is described at dx.doi.org/10.17504/protocols.io.xbgfijw.

### ****Sample and variant level quality control****

#### The Finrisk Study

In the Finrisk sample level quality control (QC) we excluded 890 samples with an ambiguous sex, a genotype missingness of more than 5% or excess heterozygosity (beyond ±4 standard deviation units from the mean or of non-European ancestry). An additional 2,212 samples were excluded due to relatedness. During the Finrisk data variant-wise QC we excluded variants with a missingness greater than 2%, Hardy-Weinberg *P* < $1\times{10}^{-6}$, a minor-allele count less than 3 and those located in the MHC region located in chromosome 6p21 or in low complexity regions of the genome. An additional 757 samples were excluded due to missing phenotype information. In the remaining 23,435 samples we filtered the high-confident PTVs within a minor-allele frequency (MAF) range of 0.1% and 5% using LOFTEE[6] (www.github.com/konradjk/loftee) and PLINK[7], version v1.90b3.45 (www.cog-genomics.org/plink/1.9/), which left us with 1,377 variants to analyze.

#### The FinnGen Study

The FinnGen genotypes first underwent basic variant and sample-level QC. Variants with a call rate less than 97%, and samples with genotyping missingness greater than 5% or with ambiguous sex were removed. Sample mix ups and extraneous duplicate copies of lower genotyping quality were manually removed. Then individuals with an ambiguous genotype-determined sex (F-score > 0.3), a genotyping success rate below 95%, excess heterozygosity (more than 4 standard deviation units from the mean or non-European ancestry), outside the population structure as specified by multidimensional scaling (maximum of five iterations) or by the first two principal components (beyond 4 standard deviation units from the mean with a maximum of five iterations) or contaminated samples (pihat linkage ≥ 0.1 with at least 14 samples) were removed. These steps were iterated through until all samples met all the criteria.

##### Genetic sample outlier detection

We performed population outlier detection using principal component analysis (PCA) and a Bayesian algorithm using 41,678 independent and common variants with a high genotype probability and low missingness according to the following filters:

- Autosomal chromosomes,
- Variants with a genotype information score ≥ 0.95,
- Variants with a missingness ≤ 0.01 (according to GP [genotype probability])
- Variants with a MAF ≥ 0.05,
- Linkage disequilibrium (LD) pruning with a window size of 500kb, step size of 50kb and a $r^{2}$ filter < 0.1

Then, these same variants were selected from the 1000 Genomes Project[8] (1000G) and merged with that of the FinnGen data. As a result of the outlier detection, 5,520 samples were removed of which 3,138 were FinnGen Study samples. This routine successfully detected all the 1000G samples with non-European and Southern European ancestry, but failed to exclude all 1000G samples with Western European origin. The cluster of Western Europeans classified as Finns was too small to perform a second round of population outlier detection, using the PCA and Bayesian algorithm routine, without detecting substructures of the Finnish population. Therefore, another PCA on the remaining FinnGen samples was performed. The European and Finnish 1000G genotypes were then projected onto the new three-dimensional space and two clusters computed. Then, we calculated the squared Mahalanobis distances of the FinnGen samples to the centroid of each cluster. Since the Mahalanobis squared distance is a sum of variables with unit variance we can see it as a sum of three independent variables and thus generate a $\chi^{2}$probability distribution with 3 degrees of freedom. In this way, we were able to calculate the probability of belonging to each cluster for every sample. Samples with more than a 0.95 probability were classed as belonging to the FinnGen cluster and were included in further analyses. As a result, 538 outliers fell below this threshold.

##### Kinship determination

To maximize the number of unrelated samples used in our association tests we first determined all the pairs of FinnGen samples up to the second degree. Next, the 588 samples with less than a 0.95 probability of belonging to the FinnGen cluster were removed. Thereafter, we used two algorithms from the network Python package (networkx.github.io/) to flag samples up to second degree kinship in the remaining FinnGen Study samples:

1. Greedy, that removes the highest degree node from the network of relations until no more links in network remain
2. Native, performed on each subgraph of the network

These two algorithms separated the samples into three sets:

1. 156,977 unrelated samples with Finnish ancestry
2. 61,980 non-duplicate samples with Finnish ancestry but who are related to the samples in the first set
3. 5,780 samples who are either of non-Finnish ancestry, are twins/duplicates related to other samples, which were excluded.

A PCA was performed for the 156,977 unrelated samples after which the 61,980 samples in the second set and were projected onto the same multidimensional space yielding population covariates for 218,957 samples.

##### Phenotype information

Of the 218,957 non-duplicate population inlier samples, we excluded 165 samples with missing minimum phenotype information or a mismatch between imputed sex and the reported sex in the registry data. Thus, a total of 218,792 samples were used for the phenome-wide association studies of the lipid-associated PTVs.

### ****Statistical analysis****

#### Primary Analyses

For the single-variant analysis on lipids, we considered a genome-wide significance (two-sided P value less than $5\times{10}^{-8}$) to be significant to account for the testing of 1,377 PTVs in 1,209 genes. For this analysis we used the Finrisk Study cohorts, whose baseline data is shown in S2 Table. The genetic association analyses were performed using PLINK[7], version v1.90b3.45 (www.cog-genomics.org/plink/1.9/), Python, version 3.6 (www.python.org), and the statsmodels Python package, version 0.8.0 (www.statsmodels.org). The conditional tests shown in S3-S6 Tables of the lipid-associated PTVs were performed with previously identified genome-wide significant variants[9-12]. Moreover, the 95% credible sets for each lipid-associated locus are shown in S7-S10 Tables.

#### Secondary and tertiary analyses

In the analyses between cardiometabolic disease risk and the lipid-associated PTVs, we considered a two-sided P value of less than 0.05 to be significant. We also tested if these PTVs were associated with the traditional non-lipid cardiometabolic risk factors: hypertension and statin medication in the FinnGen Study (S11 Table). In the phenome-wide scans we used a significance threshold of a two-sided P value less than $1.8\times{10}^{-5}$ (Bonferroni-corrected threshold for 2,683 traits) to account for statistical significance. All the 2,683 endpoints that we used in our study had at least 100 disease cases among the 218,792 samples. The tested disease case and control definitions for data freeze 5 are available at: www.finngen.fi/en/researchers/clinical-endpoints and the detailed association statistics for the tertiary analyses in S1-S2 Data. For the association analyses we used a mixed model logistic regression R/C++ package called SAIGE[13], version 0.35.8.8 (www.github.com/weizhouUMICH/SAIGE/releases/tag/0.35.8.8).

##### SAIGE null models

For computing the null model for each disease endpoint, we used age, sex, 10 principal components and the genotyping batch as covariates. To avoid convergence issues, a genotyping batch was included as a covariate for an endpoint if the batch contained at least 10 cases and controls. One genotyping batch was not included as a covariate in the model to avoid the saturation of covariate values. We excluded the AxiomGT1_b16 genotyping batch as it was not enriched for any particular disease endpoints.

For calculating the genetic relationship matrix (GRM), we used the genotype dataset where genotypes with GP < 0.95 where set as missing. Only variants with an IMPUTE2 information score > 0.95 in all genotyping batches were used. Variants with more than 3% missing genotypes or a MAF below 1% were excluded. The remaining variants were LD-pruned with a 1Mb window and r^2^ < 0.1. These operations resulted in a set of 58,702 well-imputed and non-rare variants for computing the GRM. The SAIGE options in place for computing the GRM were:

- LOCO false
- numMarkers 30
- traceCVcutoff 0.0025
- ratioCVcutoff 0.001.

## ****References****

1. Vartiainen E, Laatikainen T, Peltonen M, Juolevi A, Mannisto S, Sundvall J, et al. Thirty-five-year trends in cardiovascular risk factors in Finland. Int J Epidemiol. 2010;39(2):504-18. Epub 2009/12/05. doi: 10.1093/ije/dyp330. PubMed PMID: 19959603.

2. Goldstein JI, Crenshaw A, Carey J, Grant GB, Maguire J, Fromer M, et al. zCall: a rare variant caller for array-based genotyping: genetics and population analysis. Bioinformatics. 2012;28(19):2543-5. Epub 2012/07/31. doi: 10.1093/bioinformatics/bts479. PubMed PMID: 22843986; PubMed Central PMCID: PMCPMC3463112.

3. Loh PR, Palamara PF, Price AL. Fast and accurate long-range phasing in a UK Biobank cohort. Nat Genet. 2016;48(7):811-6. Epub 2016/06/09. doi: 10.1038/ng.3571. PubMed PMID: 27270109; PubMed Central PMCID: PMCPMC4925291.

4. Howie BN, Donnelly P, Marchini J. A flexible and accurate genotype imputation method for the next generation of genome-wide association studies. PLoS Genet. 2009;5(6):e1000529. Epub 2009/06/23. doi: 10.1371/journal.pgen.1000529. PubMed PMID: 19543373; PubMed Central PMCID: PMCPMC2689936.

5. Browning BL, Browning SR. Genotype Imputation with Millions of Reference Samples. Am J Hum Genet. 2016;98(1):116-26. Epub 2016/01/11. doi: 10.1016/j.ajhg.2015.11.020. PubMed PMID: 26748515; PubMed Central PMCID: PMCPMC4716681.

6. Lek M, Karczewski KJ, Minikel EV, Samocha KE, Banks E, Fennell T, et al. Analysis of protein-coding genetic variation in 60,706 humans. Nature. 2016;536(7616):285-91. Epub 2016/08/19. doi: 10.1038/nature19057. PubMed PMID: 27535533; PubMed Central PMCID: PMCPMC5018207.

7. Chang CC, Chow CC, Tellier LC, Vattikuti S, Purcell SM, Lee JJ. Second-generation PLINK: rising to the challenge of larger and richer datasets. Gigascience. 2015;4:7. Epub 2015/02/28. doi: 10.1186/s13742-015-0047-8. PubMed PMID: 25722852; PubMed Central PMCID: PMCPMC4342193.

8. Genomes Project C, Abecasis GR, Altshuler D, Auton A, Brooks LD, Durbin RM, et al. A map of human genome variation from population-scale sequencing. Nature. 2010;467(7319):1061-73. Epub 2010/10/29. doi: 10.1038/nature09534. PubMed PMID: 20981092; PubMed Central PMCID: PMCPMC3042601.

9. Klarin D, Damrauer SM, Cho K, Sun YV, Teslovich TM, Honerlaw J, et al. Genetics of blood lipids among ~300,000 multi-ethnic participants of the Million Veteran Program. Nat Genet. 2018;50(11):1514-23. Epub 2018/10/03. doi: 10.1038/s41588-018-0222-9. PubMed PMID: 30275531; PubMed Central PMCID: PMCPMC6521726.

10. Liu DJ, Peloso GM, Yu H, Butterworth AS, Wang X, Mahajan A, et al. Exome-wide association study of plasma lipids in >300,000 individuals. Nat Genet. 2017;49(12):1758-66. Epub 2017/10/31. doi: 10.1038/ng.3977. PubMed PMID: 29083408; PubMed Central PMCID: PMCPMC5709146.

11. Lu X, Peloso GM, Liu DJ, Wu Y, Zhang H, Zhou W, et al. Exome chip meta-analysis identifies novel loci and East Asian-specific coding variants that contribute to lipid levels and coronary artery disease. Nat Genet. 2017;49(12):1722-30. Epub 2017/10/31. doi: 10.1038/ng.3978. PubMed PMID: 29083407; PubMed Central PMCID: PMCPMC5899829.

12. Willer CJ, Schmidt EM, Sengupta S, Peloso GM, Gustafsson S, Kanoni S, et al. Discovery and refinement of loci associated with lipid levels. Nat Genet. 2013;45(11):1274-83. Epub 2013/10/08. doi: 10.1038/ng.2797. PubMed PMID: 24097068; PubMed Central PMCID: PMCPMC3838666.

13. Zhou W, Nielsen JB, Fritsche LG, Dey R, Gabrielsen ME, Wolford BN, et al. Efficiently controlling for case-control imbalance and sample relatedness in large-scale genetic association studies. Nat Genet. 2018;50(9):1335-41. Epub 2018/08/15. doi: 10.1038/s41588-018-0184-y. PubMed PMID: 30104761; PubMed Central PMCID: PMCPMC6119127.
