# Supplementary figures and images for: ANGPTL8 protein-truncating variant associated with lower serum triglycerides and risk of coronary disease
Source: PLoS Genet. 2021 Apr 28;17(4):e1009501. doi: 10.1371/journal.pgen.1009501 (PMC8109807; doi:10.1371/journal.pgen.1009501)

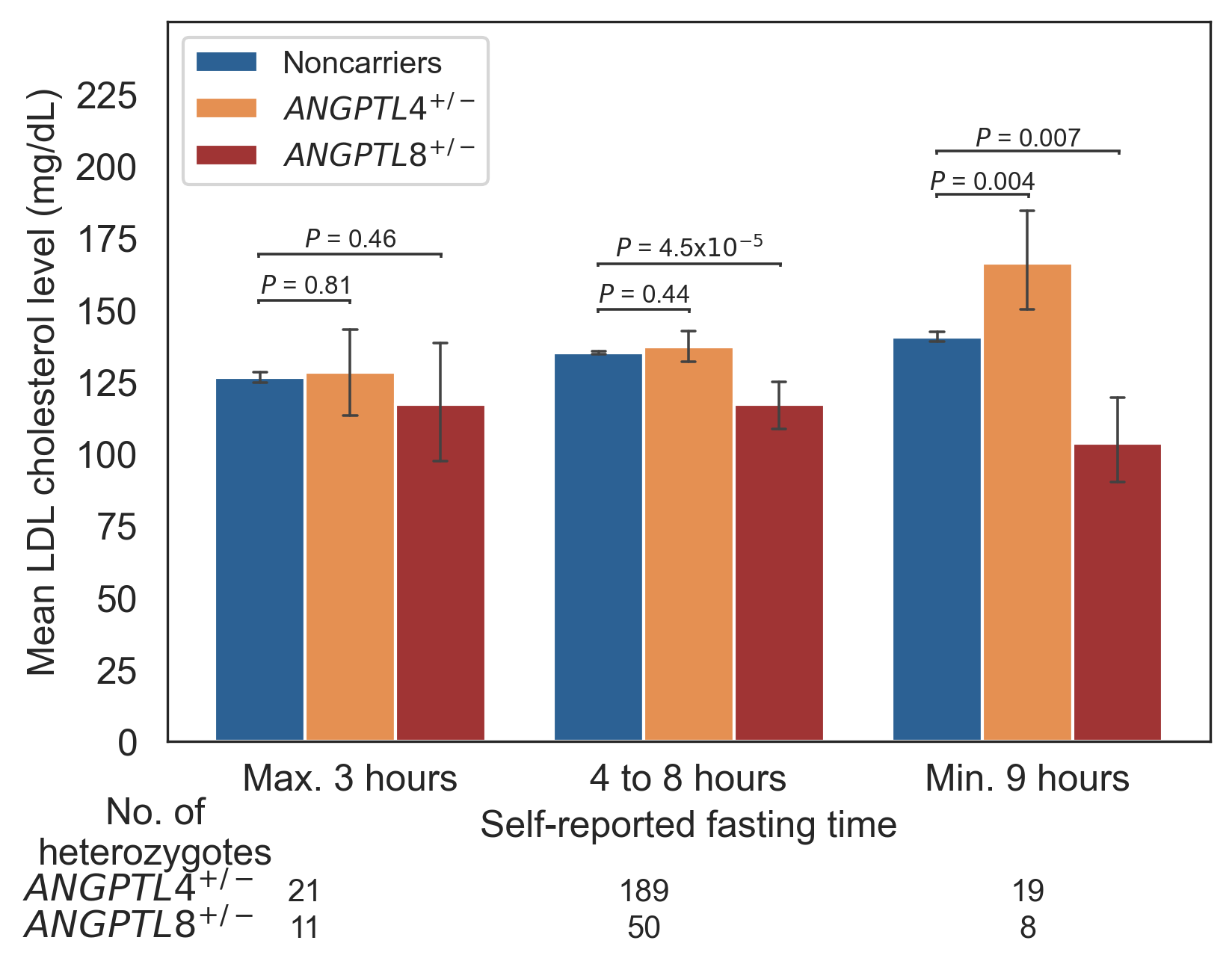

Supplement: S1 Fig — a The figure shows the mean plasma LDL cholesterol level by ANGPTL4 and ANGPTL8 PTV carrier status with respect to self-reported fasting time. The number of ANGPTL4 and ANGPTL8 heterozygotes for each fasting time interval are reported below the fasting time legend. The points indicate means and the error bars 95% confidence intervals. The P values are for the two-sided Welch’s t-test between triglyceride levels of noncarriers and heterozygotes. LDL cholesterol levels of individuals with lipid-lowering therapy were divided by 0.7. (TIFF) [file pgen.1009501.s017.tiff]
